# Discovery of novel selective PI3Kγ inhibitors through combining machine learning-based virtual screening with multiple protein structures and bio-evaluation

**DOI:** 10.1016/j.jare.2021.04.007

**Published:** 2021-04-20

**Authors:** Jingyu Zhu, Kan Li, Lei Xu, Yanfei Cai, Yun Chen, Xinling Zhao, Huazhong Li, Gang Huang, Jian Jin

**Affiliations:** a School of Pharmaceutical Sciences, Jiangnan University, Wuxi, Jiangsu 214122, China; b Institute of Bioinformatics and Medical Engineering, School of Electrical and Information Engineering, Jiangsu University of Technology, Changzhou 213001, China; c School of Biotechnology, Jiangnan University, Wuxi, Jiangsu 21412 2, China; d Shanghai Key Laboratory of Molecular Imaging, Shanghai University of Medicine and Health Sciences, Shanghai 201318, China

**Keywords:** PI3Kγ, Selective inhibitor, Hematologic malignancies, Virtual screening, Molecular dynamics simulation, JN-KI3, PI3K, Phosphoinositide 3-kinase, RTK, receptor tyrosine kinases, GPCR, G protein-coupled receptors, CADD, computer-aided drug design, VS, virtual screening, MD, molecular dynamics, PDB, protein data bank, RMSD, root-mean-squared-deviation, SP, standard precision, XP, extra precision, DS3.5, discovery studio 3.5, NBC, naive Bayesian classifier, ROC, receiver operations characteristic, AUC, area under receiver operations characteristic curve, REOS, rapid elimination of swill, Badapple, bioactivity data associative promiscuity pattern learning engine, SMILES, simplified molecular input line entry specification, ADMET, absorption, distribution, metabolism, excretion, and toxicity, DMEM, Dulbecco’s Modified Eagle Medium, IMDM, Iscove’s Modified Dulbecco’s Medium, FBS, fetal bovine serum, SD, standard deviation, AKT, protein kinase B, PAGE, polyacrylamide gel electrophoresis, RMSF, root-mean-squared-fluctuation, MM/GBSA, molecular mechanics/generalized born surface area, PAINS, pan-assay interference compounds, PSA, primary screening assays, CDRA, confirmatory dose–response assays, PARP, poly ADP-ribose polymerase, H-bond, hydrogen bond, Ionic, ionic interactions, Water Bridge, hydrogen bonds through water molecular bridge

## Abstract

**Introduction:**

Phosphoinositide 3-kinase gamma (PI3Kγ) has been regarded as a promising drug target for the treatment of various diseases, and the diverse physiological roles of class I PI3K isoforms (α, β, δ, and γ) highlight the importance of isoform selectivity in the development of PI3Kγ inhibitors. However, the high structural conservation among the PI3K family makes it a big challenge to develop selective PI3Kγ inhibitors.

**Objectives:**

A novel machine learning-based virtual screening with multiple PI3Kγ protein structures was developed to discover novel PI3Kγ inhibitors.

**Methods:**

A large chemical database was screened using the virtual screening model, the top-ranked compounds were then subjected to a series of bio-evaluations, which led to the discovery of JN-KI3. The selective inhibition mechanism of JN-KI3 against PI3Kγ was uncovered by a theoretical study.

**Results:**

49 hits were identified through virtual screening, and the cell-free enzymatic studies found that JN-KI3 selectively inhibited PI3Kγ at a concentration as low as 3,873 nM but had no inhibitory effect on Class IA PI3Ks, leading to the selective cytotoxicity on hematologic cancer cells. Meanwhile, JN-KI3 potently blocked the PI3K signaling, finally led to distinct apoptosis of hematologic cell lines at a low concentration. Lastly, the key residues of PI3Kγ and the structural characteristics of JN-KI3, which both would influence γ isoform-selective inhibition, were highlighted by systematic theoretical studies.

**Conclusion:**

The developed virtual screening model strongly manifests the robustness to find novel PI3Kγ inhibitors. JN-KI3 displays a specific cytotoxicity on hematologic tumor cells, and significantly promotes apoptosis associated with the inhibition of the PI3K signaling, which depicts PI3Kγ as a potential target for the hematologic tumor therapy. The theoretical results reveal that those key residues interacting with JN-KI3 are less common compared to most of the reported PI3Kγ inhibitors, indicating that JN-KI3 has novel structural characteristics as a selective PIK3γ inhibitor.

## Highlights

• Virtual screening based on machine learning with multiple proteins was developed.
• Discovery of a novel PI3Kγ inhibitor integrating virtual screening and bio-assays.
• JN-KI3 selective inhibit PI3K*γ* enzymatic activity and hematologic malignancies.
• The selective *γ*-inhibition mechanism of JN-KI3 was highlighted using MD simulation.

## Graphical abstract

**Figure:**
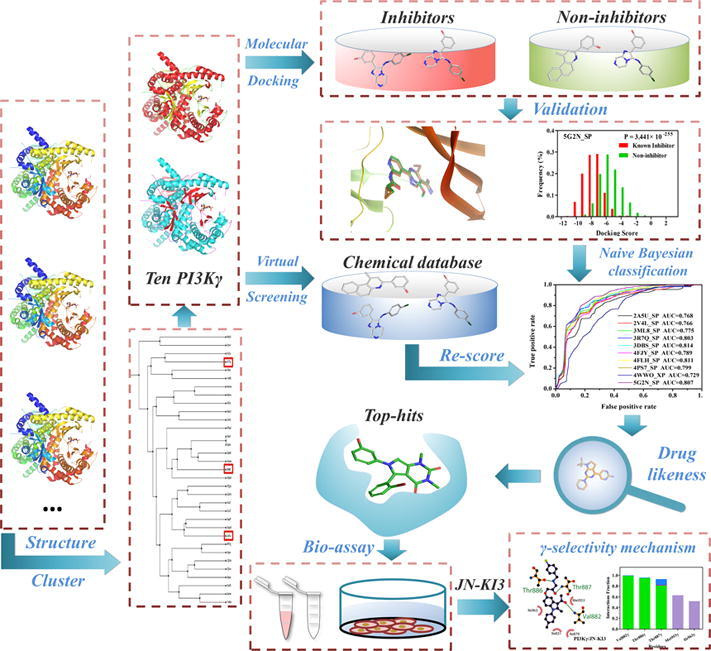

## Introduction

The extensive research conducted over the past decades has implicated the role of various cell signaling events in numerous malignant, inflammatory, autoimmune, and cardiovascular diseases, out of which the phosphoinositide 3-kinase (PI3K) signaling is one of the most relevant pathways because of its various vital functions, such as cell survival, proliferation, differentiation, and motility [1]. PI3Ks belong to a family of lipid kinases which catalyze the phosphorylation of the inositol ring of phosphoinositide and then transduce the signals as secondary messengers [2]. PI3Ks are divided into three different classes (I, II, and III) based on their primary structures and the preference of substrates [3]. Comparing to the other two classes, class I PI3Ks have been extensively and exhaustively studied. Class I PI3Ks are consist of two different subclasses, class IA and class IB, according to their signaling pathways and regulating proteins [4]. There are three PI3K isoforms, PI3Kα, PI3Kβ, and PI3Kδ, belonging to class IA, and PI3Kγ is the only member of class IB. Class IA are activated by receptor tyrosine kinases (RTKs) and other tyrosine kinase coupled receptors [5], [6], [7], while class IB accepts the signaling from G protein-coupled receptors (GPCRs) through the interaction between PI3Kγ regulatory subunit and the β-subunits of GPCRs [8], [9], [10]. A large amount of research has proved that the Class I PI3Ks play multiple crucial roles in a wide variety of cellular processes [11], [12], [13], and that makes PI3K as a promising drug target for the treatment of cancers, inflammations, and immune disorders [3], [4], [5], [6], [7]. Nowadays, four PI3K inhibitors have been pushed into the market, namely, Idelalisib in 2014 (PI3Kδ inhibitor) [14], [15], Copanlisib in 2017 (pan-PI3K inhibitor) [16], Duvelisib in 2018 (PI3Kδ/γ duel inhibitor) [17] and Alpelisib in 2019 (PI3Kα inhibitor) [18], [19]. These achievements greatly stimulated the development of novel PI3K inhibitors, especially PI3K selective inhibitors.

**Fig. 1:**
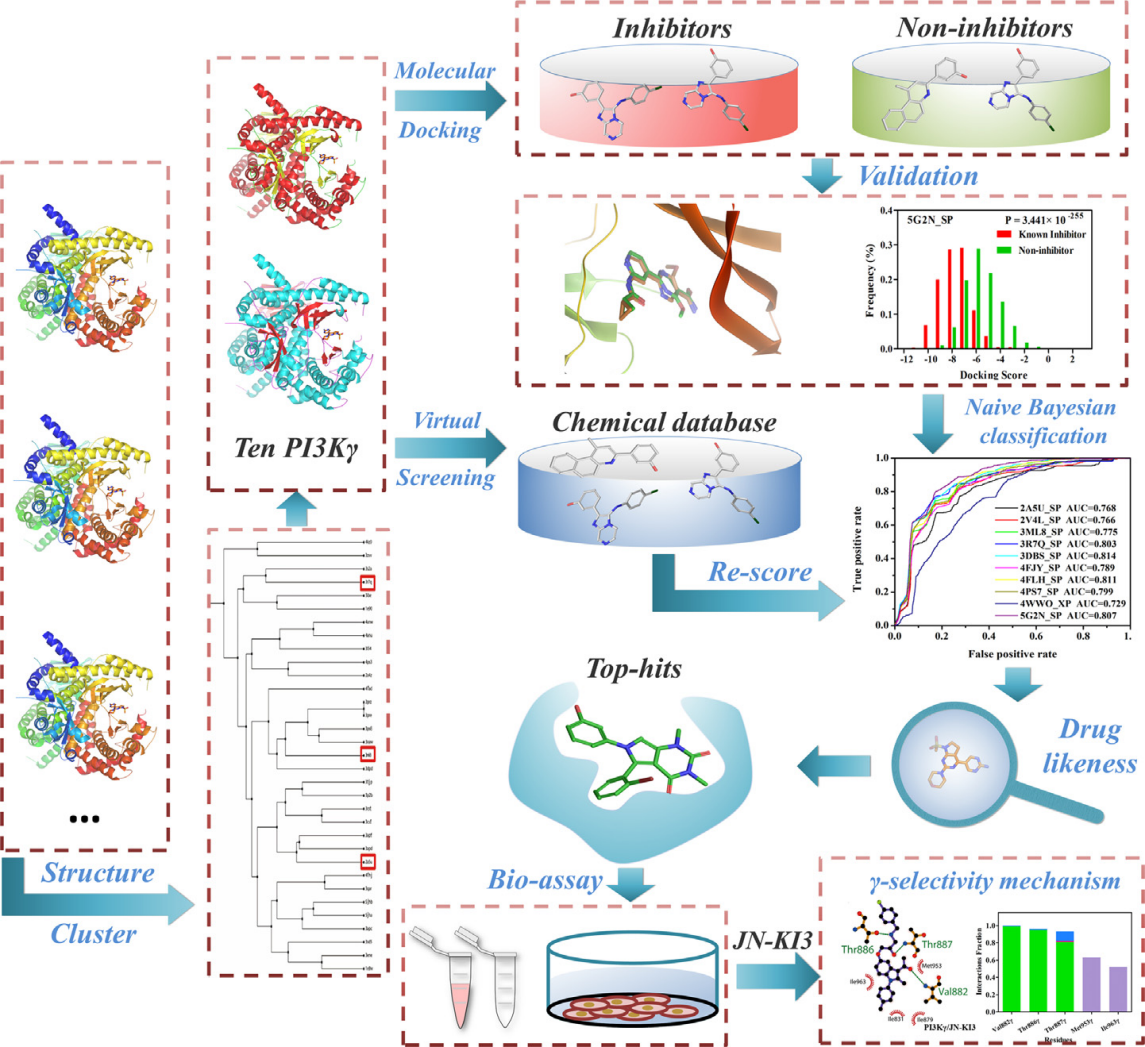
The workflow of this study.

Among the four isoforms, PI3Kγ has been attracted more and more attention for its restricted expression in the hematopoietic systems, especially in leukocytes [20]. Thus, PI3Kγ is considered as a valuable target to treat not only advanced solid tumors, leukemia and lymphoma, but also inflammatory and autoimmune diseases [21], [22], [23]. However, the high structural and sequential homology of these four PI3K isoforms is a huge hinder to acquire specific PI3Kγ inhibitor [24]. Some selective PI3Kγ inhibitors have been discovered with experimental methods and almost all of those were identified through screening against a broad panel of diverse kinases for each chemical scaffold [22], [25], [26]. It is known that these traditional experimental approaches are expensive and time-consuming [27]. Besides, it is reported that in the ATP-binding pocket of PI3Kγ, the side chains of the residues distinguished from other isoforms are always rotated away from the pocket [24]. The conventional methods are difficult to reveal these structural features at a molecular level. Therefore, computer-aided drug design (CADD) is an appropriate selection to aid in the discovery or design of PI3Kγ inhibitors with a high degree of selectivity [28], [29], [30]. Thus, in this present study, a combined approach integrated bioactive evaluation and computer simulation was conducted to discover novel PI3Kγ inhibitors. The whole workflow was given in Fig. 1. First, a machine learning-virtual screening (VS) model based on molecular docking with multi-PI3Kγ conformations was built to screen the ChemDiv database, and some top-ranked inhibitors were purchased and submitted to bioassays. As a result, some potential PI3Kγ selective inhibitors were identified. Particularly, JN-KI3 exhibits a high degree of PI3Kγ-selective inhibition and specific anti-proliferative activity against hematologic malignancies. In the end, a systematic computational analysis utilizing molecular docking and molecular dynamic (MD) simulations was performed to study the selective-PI3Kγ inhibition mechanism of JN-KI3.

## Material and methods

### Construction of VS model

#### Preparation of PI3Kγ complexes

The workflow of the VS protocol is illustrated in Fig. 1. A total of 87 crystallographic structures of PI3Kγ-inhibitor complexes were gathered and retrieved from the RCSB protein data bank (PDB, **Table S1** in the supplementary information) [31]. All PI3Kγ protein structures were prepared with *Protein Preparation Wizard* module of Schrödinger 9.0 to remove crystallographic waters, add hydrogen atoms, assign partial charges with the OPLS-2005 force fields [32], assign the protonated state. The missing side chains and loops were added using *Prime* module, and last, all protein structures were minimized until the root-mean-squared-deviation (RMSD) reached a maximum value of 0.3 Å, and other parameters were set as default. Then the residues within 10 Å of each co-crystallized ligand were reserved and structurally aligned with *STAMP* algorithm in VMD [33]. Afterward, the *phylogenetic tree* module in VMD was employed to calculate the phylogenetic tree based on the RMSD values and the result was illustrated in Fig. S1**,** all of them were divided into 10 categories and the complex with the highest resolution within each category were chosen and finally 10 representative complexes were selected to further docking simulation. Firstly, the “docking power” of these chosen complexes was evaluated with following steps: the co-crystallized ligands were redocked into the active binding pocket by Glide/SP (standard precision) and Glide/XP (extra precision) molecular docking methods, and then the RMSD between the docking results and the original conformations of inhibitors were calculated. Generally speaking, RMSD ≤ 2.0 Å represents the criterion of successful docking prediction [34].

#### Inhibitor/non-inhibitor validation set

In order to evaluate the “screening power” of the 10 complexes with the docking module, which represents the ability to distinguish the known PI3Kγ inhibitors from non-inhibitors, 800 PI3Kγ inhibitors, as an “inhibitor set”, with definite biological activity (IC_50_ < 10 μM) were extracted from BindingDB database [35] using *Find Diverse Molecules* module of Discovery Studio 3.5 (DS3.5) software. Besides, 16,000 compounds were randomly selected from ChemDiv compound library with the *Find Diverse Molecules* in DS 3.5 to construct a “decoy set”, the ratio between inhibitors and decoys was 1:20. Both “inhibitor set” and “decoy set” were minimized under OPLS-2005 force field [2] using the *LigPrep* module of Schrödinger 9.0 with the default parameters set.

#### Validation of molecular docking

10 prepared PI3Kγ complex were then submitted to perform molecular docking. the *Receptor Grid Generation* module of Schrödinger 9.0 was employed to generate a bounding box of size 10 Å × 10 Å × 10 Å within each PI3Kγ structure, and the center of each box was defined through the co-crystallized ligand in the ATP binding site. And all prepared compounds in the validation set were then docked into the ATP binding site of these PI3Kγ structures, and all docking poses were scored by the SP and XP scoring modes. The best binding pose of each compound was saved for the following analysis, and Student’s *t*-test was used to measure the difference in the distribution of docking scores between inhibitor and non-inhibitors.

#### Integrated docking with multiple PI3Kγ proteins

Consequently, a machine learning approach, naive Bayesian classification (NBC), based on these 10 PI3Kγ structures was employed to evaluate the screening accuracy. The docking scores calculated by the *Glide* docking module were used as the independent variable (X), and 1/0 (1 for PI3Kγ inhibitor and 0 for non-inhibitor) was used as the response variable (Y). Then, the predicting classifiers for VS model were generated following the *Create Bayesian Model* protocol in DS 3.5, and the ligand enrichment was determined through the area under ROC (receiver operating characteristic curve) value (AUC). The AUC represents the relationship between the true positive rate (active inhibitors) and the false positive rate (decoys), and a high AUC value indicates that the developed model contains a strong power to discriminate the positive inhibitors from the negative non-inhibitors [36].

#### Multi-conformational virtual screening in a sequential manner

Firstly, about 2,000,000 compounds from ChemDiv database were docking into the binding site of 10 PI3Kγ structures with the preferred docking precision, respectively, and 10,000 top-ranked compounds were retained. And then, the best Bayesian classifier was then utilized to re-score these 10,000 compounds, and 2,000 compounds with top Bayesian scores were reserved. Afterward, these compounds were assessed the drug-likeness by *Filter by Lipinski and Veber Rules* module in DS3.5 [37] and the *rapid elimination of swill (REOS) rules* in Canvas [38], and 200 compounds were selected. Besides, to maintain structure diversity of screening compounds, the 200 compounds were structurally clustered through the *Find Similar Molecules by Fingerprints* module in DS3.5, the compounds with similarity coefficients higher than 0.8 were clustered into the same group. And then, 100 compounds with various scaffolds were chosen. The 2D-interactions patterns between these 100 compounds and PI3Kγ following illustrated by the *Ligand Interactions* module of Schrödinger. And the compounds interacting with the “hot residues” within the ATP binding pocket, which were identified by our previous work [39], [40], were kept. Finally, the top 49 compounds with required interactions were selected as pro-hits for the following biological evaluations.

### Promiscuity and ADMET assessment

The promiscuity of JN-KI3 was investigated by two online programs, Badapple [41] (bioactivity data associative promiscuity pattern learning engine, http://pasilla.health.unm.edu/tomcat/badapple/badapple) and Hit Dexter 2.0 [42] (https://acm.zbh.uni-hamburg.de/hitdexter/). For both tools, the SMILES (simplified molecular input line entry specification) format of JN-KI3 was entered into the input box and the results would be generated automatically. In addition, the ADMET (absorption, distribution, metabolism, excretion, and toxicity) properties were predicted using *QikProp* module of Schrödinger. This module was carried out in normal mode and predicted principal descriptors and physicochemical properties of JK-KI3.

### Bio-assay evaluation

#### PI3K kinases activity in cell-free assays

The PI3K inhibitory activities of these pro-hits were all determined by ADP-Glo assay (Promega) [39]. For the initial screening, the compositions and optimized concentrations of the assay were listed as below: PI3Kγ 0.625 μg/mL, PIP2:PS 50 μg/mL, compounds 10 μM, ATP 25 μM, IPI-145 and LY294002 [43] were used as positive controls. For the JN-KI3 kinases activity assays, the compositions and optimized concentrations were listed as follows: PI3Kα 0.625 μg/mL, PI3Kβ 0.625 μg/mL, PI3Kδ 0.625 μg/mL, PI3Kγ 0.625 μg/mL, ATP 25 μM, PIP2:PS 50 μg/mL, PI-103 and LY294002 were used as positive controls. JN-KI3 and all positive compounds were serially diluted into required concentrations. In all cases, except for the control wells and background wells, the solution of PI3K enzymes was incubated with testing compounds in 384 plate for 45 min. 2.5 μL ATP/substrate mixture was added into the wells to initiate kinase reactions and incubated at room temperature for 1 h. Then, the reactions were stopped by addition of 5 μL ADP-Glo reagent and incubation for 120 min at room temperature. Before reading the luminescence signals of each well, 10 μL kinase detection buffer was added and incubated for 30 min at room temperature.

#### Cell lines and cell culture

The human cancer lines, MDA-MB-231, Patu8988, HepG2, A2780, LOVO, H1299, A549, HCT-8, SW480, 3AO, and MGC-803 were saved by the lab of Dr. Jian Jin, Jiangnan University. MCF-7 was purchased from the American Type Culture Collection (ATCC). Human malignant blood tumor cells, HL-60, U266, OPM2, OCI-My5, RPMI-8226, NCI-H929, Jurkat, LP-1, CCRF-CEM, K562, and U937, were provided by Dr. Xinliang Mao, Soochow University. MDA-MB-231, Patu8988, HepG2, A2780, LOVO, H1299, A549, HCT-8, SW480, 3AO, MGC-803, and MCF-7 were cultured in Dulbecco’s Modified Eagle Medium (DMEM, Gibco); U266, RPMI-8226, OPM2, OCI-My5, NCI-H929 and LP-1 were cultured in Iscove’s Modified Dulbecco’s Medium (IMDM. Gibco). The rest of cell lines were cultured in RPMI-1640 medium (Gibco). All mediums were supplemented with 10% fetal bovine serum (FBS, Gibco), 100 μg/mL penicillin and streptomycin, and all cells were maintained in a cell incubator with 5% CO_2_ at 37℃.

#### Cell proliferation assay

All cells were incubated in 96-well culture plates with 100 μL medium in each well. The concentration of solid cells (MDA-MB-231, MCF-7, Patu8988, HepG2, A2780, 3AO, LOVO, HCT-8, SW480, A549, H1299, and MGC-803) was 3,000/well and the concentration of suspension cells (HL-60, U266, OPM2, OCI-My5, RPMI-8226, NCI-H929, Jurkat, LP-1, CCRF-CEM, K562, and U937) was 7,500/well. Cells were then treated with 5 μL compounds and positive inhibitors, IPI-145 and LY294002, diluted in the required concentration for 72 h. The viability of the cancer cells was evaluated by Thiazolyl Blue (MTT) assay. 10 μL of 5 mg/mL MTT was added to each well and the plate was incubated at 37℃ for 4 h. Then, 100 μL MTT buffer was added to each well. After incubating the plate overnight, the absorbance of each well was measured at 550 nm by a microplate reader. All data were normalized to control groups (5 μL DMSO) and converted into the percent of living cells. All results were represented as the mean and standard deviation (SD) of the three independent measurements.

#### Western Blot assay

K562 and RPMI-8226 cells were cultured in 6-well plates with 1 × 10^6^ cells per well and then treated with various concentrations of JN-KI3: 0, 2.5, 5, 10, and 20 μM. After 24 h, the cells of each well were collected and washed by PBS 3 times. The sedimentary cells were then incubated in an ice bath with 80 μL RIPA lysis buffer (Beyotime) for 20 min and then centrifuged at 12,000 rpm for 20 min. The supernatant was collected and its total concentration of protein was quantified using the BCA protein assay kit (Biosharp). Loading buffer (5 ×) was added to all protein samples prepared for Western Blot assays and boiled at 100℃ for 5 min.

The inhibitory situation of PI3K/AKT (Protein kinase B) signaling pathway was then determined by Western Blot assays. The SDS-polyacrylamide gel electrophoresis (PAGE) was used to separate the proteins. All protein samples and Protein Marker (Proteintech) were loaded onto an acrylamide gel and ran for 30 min at 80 V within the staking gel and for 1 h at 100 V with the separating gel. Then, the protein was electro-transferred onto an NC membrane at 100 V for 1 h. After that, the membrane was blocked in 5% skim milk diluted in TBST (1 ×) for 2 h and then the membrane was incubated with relevant primary antibody overnight at 4℃: Phospho-AKT (Thr308) (1 : 1000, cat# 13038 T; Cell Signaling Technology), Phospho-AKT (Ser473) (1: 1000, cat# 4060 T; Cell Signaling Technology), AKT (pan) (1: 1000, cat# 4691 T; Cell Signaling Technology), caspase-3 (1: 1000, cat# sc-56053; Santa Cruz Biotechnology), PARP (1: 1000, cat# 9542S; Cell Signaling Technology). GAPDH (1: 5000, cat#60004–1-Ig; Santa Cruz Biotechnology), After 10-min washing in TBST (1 ×) for 3 times, the membrane was incubated with relevant secondary antibody at room temperature on a shaker for 1 h. And the membrane was washed for 4 times in TBST again before being imaged.

#### Flow cytometry assay

After being treated with JN-KI3 for 24 h, K562 and RPMI-8226 were stained with Annexin V-FITC/PI detection kit (Beyotime) and the apoptosis of the cells were detected using a flow cytometer (FACSCalibur, BD) following the instructions.

### Theoretical studies on the PI3Kγ selective inhibition mechanism of JN-KI3

*Docking studies.* The crystallographic structures with high resolutions of PI3Kα (PDB ID: 6PYS) [44], PI3Kβ (PDB ID: 2Y3A) [45], PI3Kδ (PDB ID: 6PYR) [44] and PI3Kγ (PDB ID: 4ANV) [46] were stretched from the RCSB PDB. All structures were prepared in the *Protein Preparation Wizard* module of Schrödinger 9.0. The 3D-structures of Cpd5, Cpd36, Cpd38, and JN-KI3 were generated by *maestro* and minimized in the *LigPrep* module. Then, a bounding box was generated on the ATP binding site of each complex with the center of crystallized ligand by using the *Receptor Grid Generation* module. Cpd5, Cpd36, and Cpd38 were docked into PI3Kγ, while JN-KI3 was docked into the binding site of each complex, all docking programs were performed using *Glide* SP method.

*MD simulations.* Four PI3Ks/JN-KI3 complexes with the best conformations generated by molecular docking were used as initial structures to perform MD simulations by *Desmond* [47]. Each complex system was immersed in a solvent model with TIP3P water molecules and the boundary of the infiltration volume was extended to 10 Å in each direction. Then, each system was neutralized with Na^+^/Cl^-^ counter ions and minimized under the OPLS-2005 force field [2]. Afterward, four complexes were submitted to MD simulations following the default NPT and NVT ensemble protocols in *Desmond* program, a 200-ns MD simulation at the pressure of 1 atm maintained by Martyn-Tobias-Klein pressure bath [48] and at the temperature of 300 K maintained by Nose- Hoover Chain thermostat [49] was performed for each system. The Smooth Particle Mesh Ewald method [50] was used to analyze the electrostatic interaction forces between the complexes. And the RMSDs of the PI3Ks protein backbones and the RMSF (root-mean-squared-fluctuation) value of each atom in JN-KI3 were calculated.

*MM/GBSA free energy calculation.* The binding free energy (ΔG_Bind_) for each system was calculated by using *Prime MM/GBSA* (Molecular Mechanics/Generalized Born Surface Area) module employing an equation:

$${\Delta G}_{\mathrm{Bind}}=G_{\mathrm{complex}}-\left(G_{\mathrm{Protein}}+G_{\mathrm{ligand}}\right)$$

Where G_complex_, G_protein_ and G_ligand_ are the prime energies of the optimized complex, free receptor and free ligand, respectively [51].

## Results and discussion

### Performance of the VS model

Accumulative studies have shown that multi-conformational virtual screening, which could mimic the induced-fit effect of the protein, in a sequential or parallel manner could be the most appropriate to balance efficiency and precision of VS than the conventional VS, which usually uses single one protein with “rigid” structure to guarantee the optimum efficiency of VS. Therefore, 87 PI3Kγ crystallographic structures from PDB were collected and then divided into 10 conformational types after the clustering analysis. The complex with the highest resolution in each class was chosen and finally 10 representative complexes were selected, namely, 2A5U (Resolution: 2.70 Å) [22], 2V4L (2.50 Å) [52], 3DBS (2.80 Å) [53], 3ML8 (2.70 Å) [54], 3R7Q (2.50 Å) [55], 4FJY (2.90 Å) [56], 4FLH (2.60 Å) [57], 4PS7 (2.69 Å) [58], 4WWO (2.30 Å) [59] and 5G2N (2.68 Å) [60] (PDB ID). In order to estimate the precision and stability of these docking model systems, the “docking power” and “screening power” were evaluated, respectively. The “docking power”, which is a vital criterion of the reliability of molecular docking, presents the accuracy of poses prediction. For each PI3Kγ complex, the crystallized ligand was re-docked into the binding site and the RMSD between the docking pose and its original conformation was calculated. The results were tabulated in Table 1**,** the RMSD of ten PI3Kγ complexes were all<2.0 Å, which means that all of them contain remarkable predicting power through both Glide/SP or Glide/XP molecular docking methods.

**Table 1:** The docking power, screening power, and crucial interactions of ten PI3Kγ crystal structures.

PDB ID	RMSD (Å)		P value		Interactions
	SP	XP	SP	XP	
2A5U	0.776	0.746	**1.612 × 10^-158^**	9.675 × 10^-128^	Asp964, Asp836
2V4L	0.789	0.779	**2.740 × 10^-193^**	3.007 × 10^-163^	Val882, Lys833
3DBS	0.830	0.816	**1.961 × 10^-227^**	3.239 × 10^-208^	Val882, Tyr867
3ML8	0.810	0.801	**4.483 × 10^-199^**	4.384 × 10^-195^	Val882, Asp964
3R7Q	0.818	0.803	**1.868 × 10^-205^**	7.619 × 10^-191^	Val882, Tyr867
4FJY	0.793	0.753	**8.318 × 10^-183^**	5.004 × 10^-125^	Val882
4FLH	0.824	0.799	**2.158 × 10-^212^**	2.889 × 10^-164^	Val882, Lys833
4PS7	0.816	0.798	**1.970 × 10^-198^**	3.452 × 10^-178^	Val882, Tyr867
4WWO	0.721	0.171	4.909 × 10^-84^	**7.920 × 10^-103^**	Val882
5G2N	0.849	0.835	**3.441 × 10^-255^**	6.537 × 10^-253^	Val88, Lys833

**Fig. 2:**
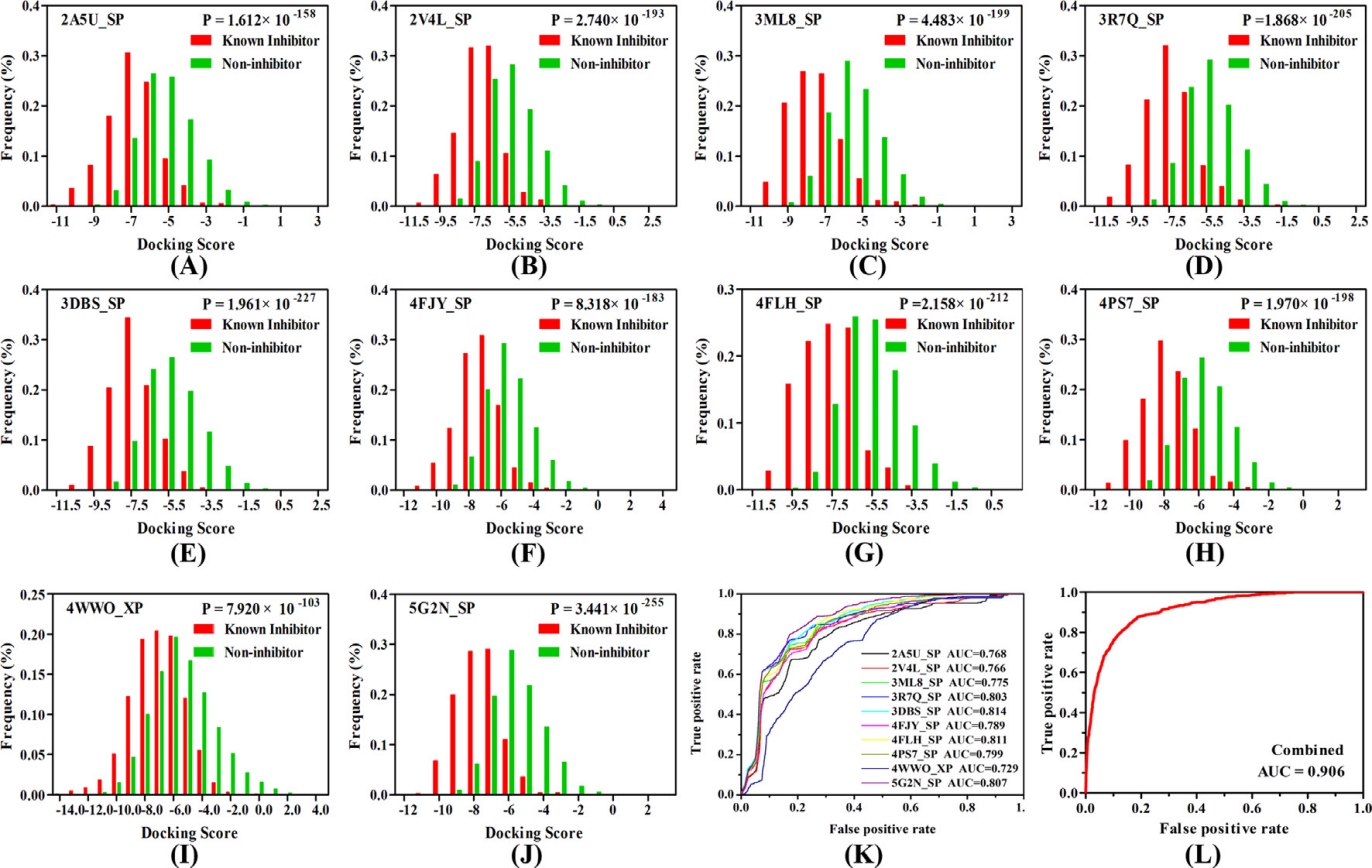
**(A-J)** Distributions of the docking scores of the inhibitor (red)/noninhibitor (green) for each PI3Kγ protein with the best discrimination power; **(K)** the ROC curve of the naive Bayesian classifier based on every single docking score; **(L)** the ROC curve of the naive Bayesian classifier based on the combined docking scores.

On the other hand, the “screening power” was investigated, which exhibits an ability of the docking methods to distinguish the active PI3Kγ inhibitors from non-inhibitors. Herein, 800 PI3Kγ inhibitors with definite bio-activity and 16,000 non-inhibitors were chosen to make up a validation set. All compounds in the dataset were all docked into ten PI3Kγ proteins with SP and XP modes, respectively. The distributions of two docking scores of each protein were illustrated, and the T-test was carried out to evaluate the discrimination capacity of each protein with P-value. As shown in Table 1, both SP and XP could accurately distinguish the inhibitors from non-inhibitors for ten PI3Kγ proteins (P-value < 0.05), but overall, the performance of SP shows better than XP in each system, except 4WWO **(**Fig. 2**)**. Take 2A5U as an example, the P-value of SP mode is 1.612 × 10^-158^, which is much<9.675 × 10^-128^, the P-value of XP. Some studies have demonstrated that even though XP is the extra precision mode of the *Glide* module and considered to be more accurate, the performance of SP is not always worse than it. Therefore, in our studied PI3Kγ systems, except 4WWO, SP mode with superior “screening power” would be adopted.

Next, the data matrix consisting of the docking scores from the above validation protocols was utilized to build the NBC model. The capability to distinguish between inhibitors and non-inhibitors for the NBC model was measured through the AUC value. Firstly, ten NBC models were constructed based on a single docking score for each complex, as summarized in Fig. 2**K**. The NBC model of 3DBS_SP (both inhibitors and non-inhibitors were docked into protein following Glide/SP protocol) shows the best predicting power and the AUC value is 0.814. The AUC value of each system is 0.768 (2A5U_SP), 0.766 (2V4L_SP), 0.775 (3ML8_SP), 0.803 (3R7Q_SP), 0.789 (4FJY_SP), 0.811 (4FLH_SP), 0.799 (4PS7_SP), 0.729 (4WWO_XP) and 0.807 (5G2N_SP), respectively (Fig. 2**K)**. Generally, the AUC value > 0.7 indicates that the model contains a satisfactory prediction power, thus a novel NBC model based on the combined docking scores of the ten PI3Kγ proteins was built up, as shown in Fig. 2**L**, the AUC value of this model is as high as 0.906, showing an excellent prediction for PI3Kγ inhibitors.

### JN-KI3 selectively inhibit PI3Kγ

**Fig. 3:**
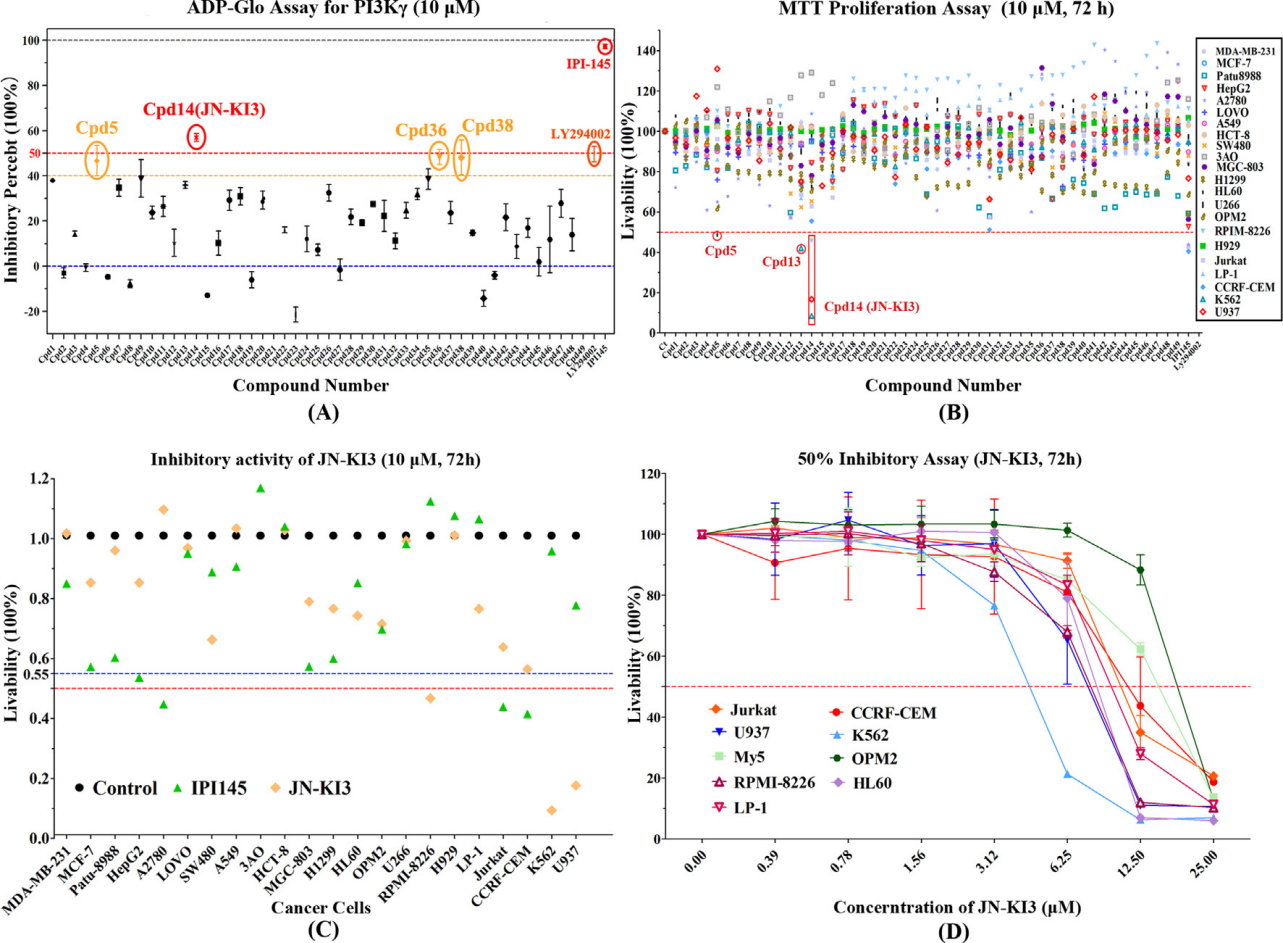
**(A)** The cell-free PI3Kγ inhibitory activities of 49 hits and two positive inhibitors, IPI-145 and LY294002; **(B)** the anti-proliferation effects towards multiple tumor cells of 49 hits and two positive inhibitors, IPI-145 and LY294002; **(C)** the anti-proliferation effects towards multiple tumor cells of JN-KI3 and IPI-145; **(D)** the anti-proliferation effects towards multiple malignant tumor cells of JN-KI3 with the gradient of concentrations.

The 49 pro-hits identified from VS were numbered as Cpd1 to Cpd49. The cell-free PI3Kγ inhibitory activities of these hits were firstly validated by ADP-Glo assay. The reported inhibitors, IPI-145 and LY294002, were tested as positive controls. The PI3Kγ-inhibitory activity of each hit was primitively validated at the concentration of 10 μM and the results were shown in Fig. 3**A**. At first glance, the marketed drug, IPI-145, showed the strongest inhibitory activity but it is encouraging that there are four hits with the inhibitory percent exceeding 50%, namely, Cpd5, JN-KI3 (Cpd14), Cpd36 and Cpd38. Among these four compounds, JN-KI3 has the most satisfying activity with almost 60% inhibitory percent to PI3Kγ, the 2D structure of JN-KI3 is illustrated in Table 2**.** The molecular docking results between these four compounds and PI3Kγ show that these compounds all form hydrogen bonds (H-bond) with Val882 of PI3Kγ (Fig. S2), Val882 have been identified as a core residue, which could form characteristic H-bond in almost all of the PI3Kγ/inhibitors complexes. Therefore, these four compounds all cause varying degrees of PI3Kγ inhibition. The alignment of the four compounds in the binding pocket shows JN-KI3 could form stronger hydrophobic interaction with PI3Kγ because of its chlorine phenyl group (Fig. S2**A**). The modeling results also reveal that JN-KI3 contains a novel scaffold different from the classical propeller-shaped core structure, like IPI-549. Then, the selective inhibition of JN-KI3 on other PI3K isoforms was evaluated and the potent pan-PI3K inhibitor, PI-103, was used as the positive control, the results summarized in Table 2. The IC_50_ value of JN-KI3 against PI3Kγ is 3,873 nM, whose inhibitory potency is near to LY294002. Though JN-KI3 shows lower bio-activity on PI3Ks than PI-103, encouragingly, it exhibits a significant specificity to PI3Kγ, as shown in Table 2, the IC_50_ values of JN-KI3 against other three isoforms are all>20,000 nM. Comparing to JN-PK1, which is an effective hit identified from our previous traditional VS work, JN-KI3 appeared not only a more significant PI3Kγ inhibitory activity, but also a higher selectivity against PI3Kγ. This result strongly proves that the VS integrated a machine learning based on multi-conformational PI3Kγ could effectively discover novel selective PI3Kγ inhibitors.

**Table 2:** The 2D-structures and the cell-free inhibitory activity values (IC_50_) of JN-KI3 and the positive reference compounds JN-PK1, PI-103, and LY294002.

Compounds	Structures	Cell-free inhibitory IC_50_ (nM)^a^			
		PI3Kγ	PI3Kα	PI3Kβ	PI3Kδ
JN-KI3		3,873	>20,000	>20,000	>20,000
JN-PK1		7,490	11,300	>20,000	>20,000
PI-103		50	3.8	10	5.1
LY294002		2,980	1,660	2,580	780

a Average of triple tests.

### The promiscuity and ADMET assessments for JN-KI3

Pan-assay interference compounds (PAINS) analysis was used to investigate how far JN-KI3 could be flagged as a PAINS compound. PAINS refer to a series of promiscuous compounds specifically binding to different macromolecular targets and leading to misleading false-positive results in experimental assays. Therein, two available promiscuous predicting algorithms, Hit Dexter 2.0 and Badapple, were utilized to examine whether JN-KI3 could be categorized as PAINS. The detailed promiscuity analysis result of JN-KI3 using Hit Dexter 2.0 is available on the website (https://nerdd.zbh.uni-hamburg.de/hitdexter/result/c1ac770f-753e-455b-8c66-a5ed2337dbfd). JN-KI3 is predicted as non-promiscuous by the PSA (primary screening assays) classifier with a probability of 0.90 and the CDRA (confirmatory dose–response assays) classifier with a probability of 1.00 at moderate confidence. Besides, JN-KI3 shows the low promiscuous probability values even at high confidence, 0.26 for PSA, and 0.17 for CDRA (Fig. S3**)**. It demonstrates that JN-KI3 would be a specific compound rather than a promiscuous one. Promiscuity screening data of Badapple are listed in Table 3. Scaf1, the indolyl group of JN-KI3, is found to have a pScore value higher than 300, accompanied with a “True” result inDrug database. The other scaffold, Scaf2, 1-phenyl-indole, presented a moderate pScore value with a “True” result inDrug database. These results suggest both promiscuity and drug-likeness probability of JN-KI3. However, no pScore was generated for Scaf3 and Scaf4, and the inDrug values are both “False”, indicating the absence of the scaffolds in the drug database.

**Table 3:** The pScore and inDrug values from Badapple data base, and the predicted ADMET properties of JK-KI3.

Compound	Scaffold Number	Scaffold Structure	pScore^a^	inDrug^b^
JN-KI3	Scaf1		370	True
	Scaf2		186	True
	Scaf3		None	False
	Scaf4		None	False

**ADMET Prediction**					

**Properties**^c^	**Value**	**Optimal range**	**Properties**	**Value**	**Optimal range**

**CNS**	1	−2 - +2	**polrz**	51.04	13.0–70.0
**logPC16**	16.27	4.0–18.0	**logPoct**	23.54	8.0–35.0
**logPw**	11.51	4.0–45.0	**logPo/w**	5.53	−2.0–6.5
**logS**	−6.31	−6.5–0.5	**CIlogS**	−6.67	−6.5–0.5
**logHERG**	−7.53	concern below −5	**PCaco**	364.52	<25 poor,>500 great
**logBB**	−0.37	−3.0–1.2	**PMDCK**	453.07	<25 poor,>500 great
**logKp**	−3.08	−8.0 - −1.0	**logKhsa**	1.05	−1.5–1.5
**PHOA**	92.20	>80% is high<25% is poor	**rtvFG**	0	0–2

a pScore values advisory: <100 (low), means no indication; 100–300 (moderate), means weak indication of promiscuity; >300 (high), means strong indication of promiscuity.

b inDrug results: true, means it was found in the drug data base; false, means not found.

c properties: CNS, predicted central nervous system activity on a −2 (inactive) to + 2 (active) scale; Polrz, predicted polarizability in cubic angstroms; logPC16, predicted hexadecane/gas partition coefficient; logPoct, predicted octanol/gas partition coefficient; logPw, predicted water/gas partition coefficient; logPo/w, predicted octanol/water partition coefficient; logS, predicted aqueous solubility; CIlogS, conformation-independent predicted aqueous solubility; logHERG, predicted IC_50_ value for blockage of HERG K^+^ channels; PCaco, predicted apparent Caco-2 cell permeability in nm/sec; logBB, predicted brain/blood partition coefficient; PMDCK, predicted apparent MDCK cell permeability in nm/sec. logKp, predicted skin permeability; logKhsa, prediction of binding to human serum albumin; PHOA, predicted human oral absorption on 0 to 100% scale; rtvFG, Number of reactive functional groups.

Besides, *in silico* ADMET of JN-KI3 were calculated to predict the pharmacochemical properties. Table 3 shows that almost all properties are within the acceptable range. For instance, all the logP values are within the optimal range, the human oral absorption (PHOA) of JN-KI3 is close to the highest recommended values,100%. All these predictions indicate that JN-KI3 exhibits optimum drug-like properties.

### JN-KI3 selectively anti-proliferate against hematologic cancer cells

At the same time, the anti-proliferative activity of each hit towards different human cancer cells was evaluated, including breast cancer cells (MDA-MB-231, MCF-7), pancreatic cancer cell (Patu8988), liver cancer cell (HepG2), ovarian cancer cells (A2780, 3AO), colon cancer cells (LOVO, HCT-8, SW480), lung cancer cells (A549, H1299), gastric cancer cell (MGC-803), and hematologic malignancies cells (HL-60, U266, OPM2, PRMI-8226, NCI-H929, Jurkat, LP-1, CCRF-CEM, K562, U937). IPI-145 and LY294002 were still used as positive controls. The anti-proliferative activity of each hit was shown as the livability of the cells. Fig. 3**B** shows that there are three hits considerably inhibiting the proliferation of some cancer cell lines. To be specific, Cpd5 could suppress the proliferation of acute promyelocytic leukemia (HL-60), Cpd13 could inhibit chronic myelogenous leukemia (K562). It is a pity that the results from the cellular experiments are not in good agreement with those of above cell-free assays, such as Cpd36 and Cpd38, which show little anti-tumor effect. But luckily, JN-KI3 show potently suppressed proliferation in some cell lines and more interestingly, JN-KI3 seems to prefer to inhibit the proliferation of hematologic cancer cell lines, such as K562 and multiple myeloma cell lines (RPMI-8226 and U937), and the inhibition effect is even stronger than IPI-145 (Fig. 3**C**). As discussed above, PI3Kγ is mainly distributed and expressed in the hematopoietic systems, that may lead to the specific inhibition against hematologic cancer cell lines. To test this hypothesis, more hematologic tumor cell lines were treated with JN-KI3, including four human leukemia cells (HL-60, Jurkat, CCRF-CEM, K562), four multiple myeloma cells (OPM2, OCI-My5, LP-1, RPMI-8226) and one lymphoma cells (U937). As shown in Fig. 3**D**, the proliferation of all cells was decreased in a concentration-dependent manner, especially K562, which show significant sensitivity to JN-KI3 at a low concentration.

### JN-KI3 induce apoptosis of hematologic cancer cells with selective inhibition on PI3K/AKT signaling pathway

**Fig. 4:**
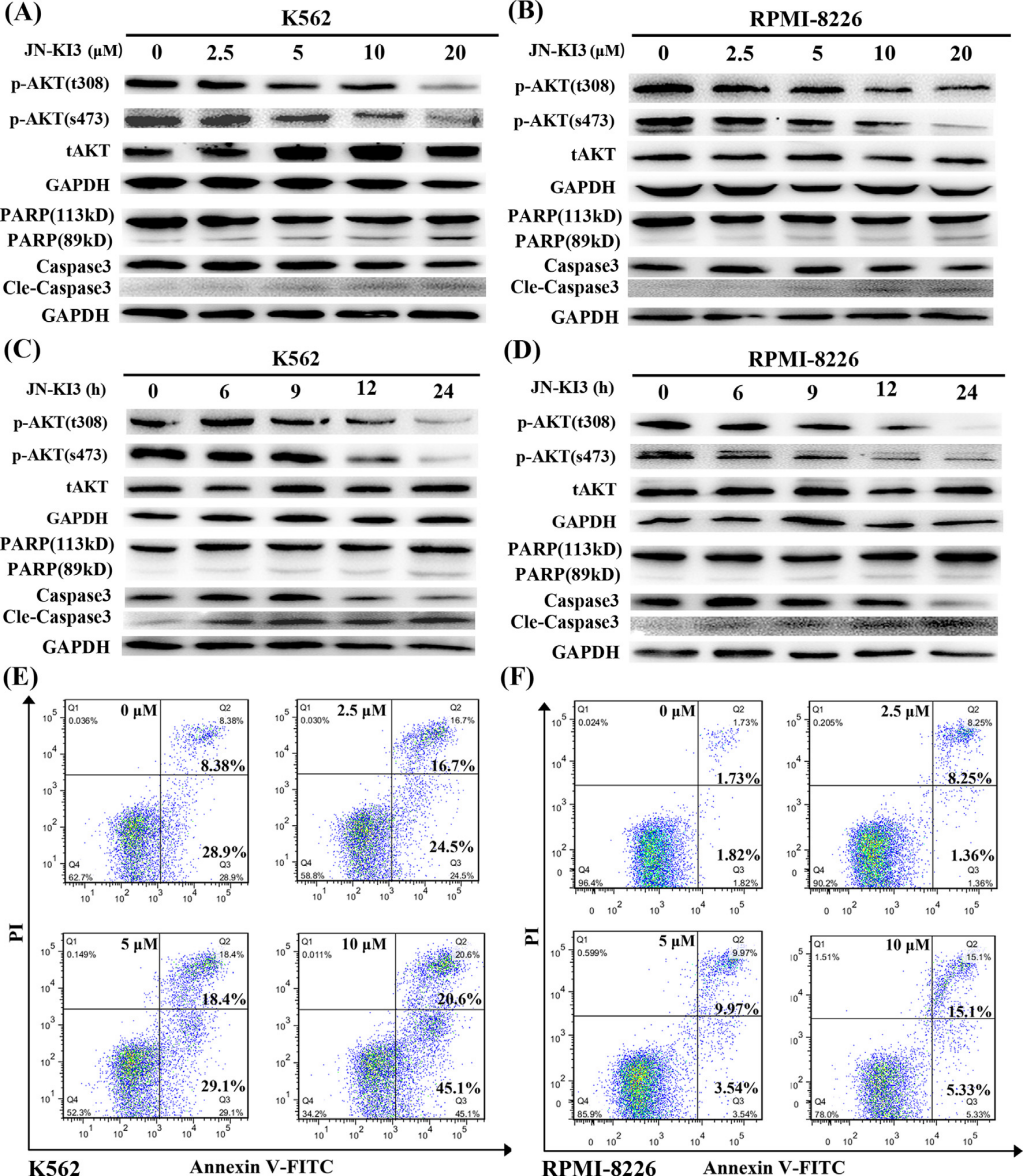
JN-KI3 inhibited PI3K/AKT signaling pathway and induced apoptosis of **(A)** K562 and **(B)** RPMI-8226 cell lines at different concentrations from 0 to 20 μM after 24 h incubation. JN-KI3 inhibited PI3K/AKT signaling pathway and induced apoptosis of **(C)** K562 and **(D)** RPMI-8226 cell lines at different times from 0 to 24 h for 20 μM concentration. JN-KI3 induced apoptosis of hematologic malignancies illustrating by flow cytometry. Apoptosis of **(E)** K562 and **(F)** RPMI-8226 cell lines treated with different concentrations of JN-KI3 (from 0 μM to 10 μM) for 24 h.

As it has been widely reported that PI3Kγ/AKT signaling pathway play central roles in regulating the survival and proliferative activities in hematologic malignancies. To investigate the inhibition of JN-KI3 to PI3K at the cellular level, immunoblotting assays were performed with the two sensitive cell lines, K562, and RPMI-8226 (8226). AKT is a node protein downstream of the PI3K signaling pathway. The inhibition of PI3Kγ kinase activity can suppress the phosphorylation of AKT at Thr308 and Ser473 sites. Thus, the inhibitory activity against PI3K of JN-KI3 at a cellular level was verified using AKT phosphorylation as a readout. As shown in Fig. 4**A & B**, after treating with the increasing concentration of JN-KI3 for 24 h, AKT phosphorylation at Thr308 and Ser473 sites in K562 and 8226 cell lines were both markedly decreased, and the inhibition was caused even at a concentration as low as 2.5 μM, while the expression of total AKT (t-AKT) was not affected. In addition, both K562 and 8226 cell lines were treated with 20 μM JN-KI3 for the indicated time periods. As shown in Fig. 4**C & D,** the phosphorylation level of AKT at both sites was significantly inhibited with the prolongation of the time of JN-KI3 action. Similarly, t-AKT was not markedly affected. All the above results indicated that JN-KI3 would effectively inhibit PI3K/AKT signaling with a concentration- and time-dependent manners in hematologic cancer cells.

Those above results suggested that JN-KI3 could specifically inhibit PI3Kγ both at the cell-free and the cellular level, and it selectively suppressed hematologic cancer cells. To further demonstrate whether JN-KI3 could induce the apoptosis of hematologic cells with the inhibition of PI3Kγ, K562 and 8226 were treated with JN-KI3 with increased duration or concentrations, and the relative levels of two apoptosis biomarkers, PARP (poly ADP-ribose polymerase) and caspase-3 were detected and the results were illustrated in Fig. 4. Caspase-3 belongs to the caspase family of cysteine proteases, which are the key mediators of cell apoptosis. Caspase-3 cleaves the death substrate PARP to a specific 89 kDa form observed during apoptosis. Fig. 4**A & B** show that after incubating with the increasing concentrations of JN-KI3, the cleavage levels of PARP and caspase-3 in K562 and 8226 cell lines were obviously increased and showing in the dependent manner of concentration. Then, a time-course study was conducted, and both K562 and 8226 treated with 20 μM JN-KI3 activated the PARP and caspase-3 following increased incubation time (Fig. 4**C & D)**. The results demonstrated that the cleavage level of Caspase-3 and PARP in both cell lines significantly increased after a short time (6 h) of incubation with JN-KI3.

To further observe the induction of apoptosis by JN-KI3, Annexin V-FITC/PI double staining was performed to K562 and 8226 treated with increasing concentrations of JN-KI3 for 24 h, and then flow cytometry was utilized to observe the apoptotic cancer cells. As shown in Fig. 4**E**, JN-KI3 could induce the apoptosis of K562 at considerably low concentration, there was 41.2%, 47.5% and 65.7% of K562 cells occurring apoptosis after treating with 2.5, 5 and 10 μM JN-KI3 for 24 h, respectively. Comparing to K562, 8226 presented moderate cellular apoptosis, about 20.43% of 8226 cells occurred definite apoptosis at a JN-KI3 concentration up to 10 μM (Fig. 4**F**). These results are good agreement with those in cell proliferation bioassay. To sum up, JN-KI3 could induce apoptosis of hematologic cancer cells in association with its potent PI3Kγ inhibition.

### Theoretical studies on the PI3Kγ selective inhibition mechanism of JN-KI3

In order to reveal the mechanism of selective inhibition of PI3Kγ by JN-KI3 at a molecular level, a computational strategy integrating molecular docking, MD simulation and free energy calculations was employed. JN-KI3 was firstly docked into the ATP-binding pocket of four PI3K isoforms and the docking scores were listed in Table 4**.** It is delighted to find that the docking scores are consistent with the experimental inhibitory activities. The docking score of PI3Kγ/JN-KI3 is −7.841 kcal/mol, which is higher than those of PI3Kα/JN-KI3 (-6.730 kcal/mol), PI3Kβ/JN-KI3 (-6.640 kcal/mol) and PI3Kδ/JN-KI3 (-6.018 kcal/mol). It roughly demonstrated that JN-KI3 has a stronger binding affinity to PI3Kγ than the other three PI3K isoforms.

**Table 4:** The docking scores and predicted binding free energies of JN-KI3/PI3Ks, and the inhibitory activities against PI3Ks of JN-KI3.

JN-KI3	PI3Kγ	PI3Kα	PI3Kβ	PI3Kδ
Docking Scores (kcal/mol)	−7.841	−6.730	−6.640	−6.018
Binding free energy (kcal/mol)	−64.981	−42.832	−36.875	−49.441
IC_50_ (μM)	3.873	＞20	＞20	＞20

To further estimate the dynamic behavior of PI3Ks after binding to JN-KI3, the MD simulations were implemented and the docking poses from molecular docking studies (Fig. S4) were served as initial structures. The backbone RMSD values of four complexes were illustrated in Fig. S5**,** which indicated each system reached equilibrium in a short time after the simulations began. When the systems were stable, MM/GBSA method was utilized to calculate the binding free energy of each system. All results are listed in Table 4. The binding free energies of PI3Kα/JN-KI3, PI3Kβ/JN-KI3, PI3Kδ/JN-KI3, and PI3Kγ/JN-KI3 are −42.832, −36.875, −49.441 and −64.981 kcal/mol, respectively. The predicted binding free energy of PI3Kγ/JN-KI3 is greatly lower than the other three systems, which suggest that JN-KI3 binds to PI3Kγ most tightly and it agrees with the above experimental and docking results.

**Fig. 5:**
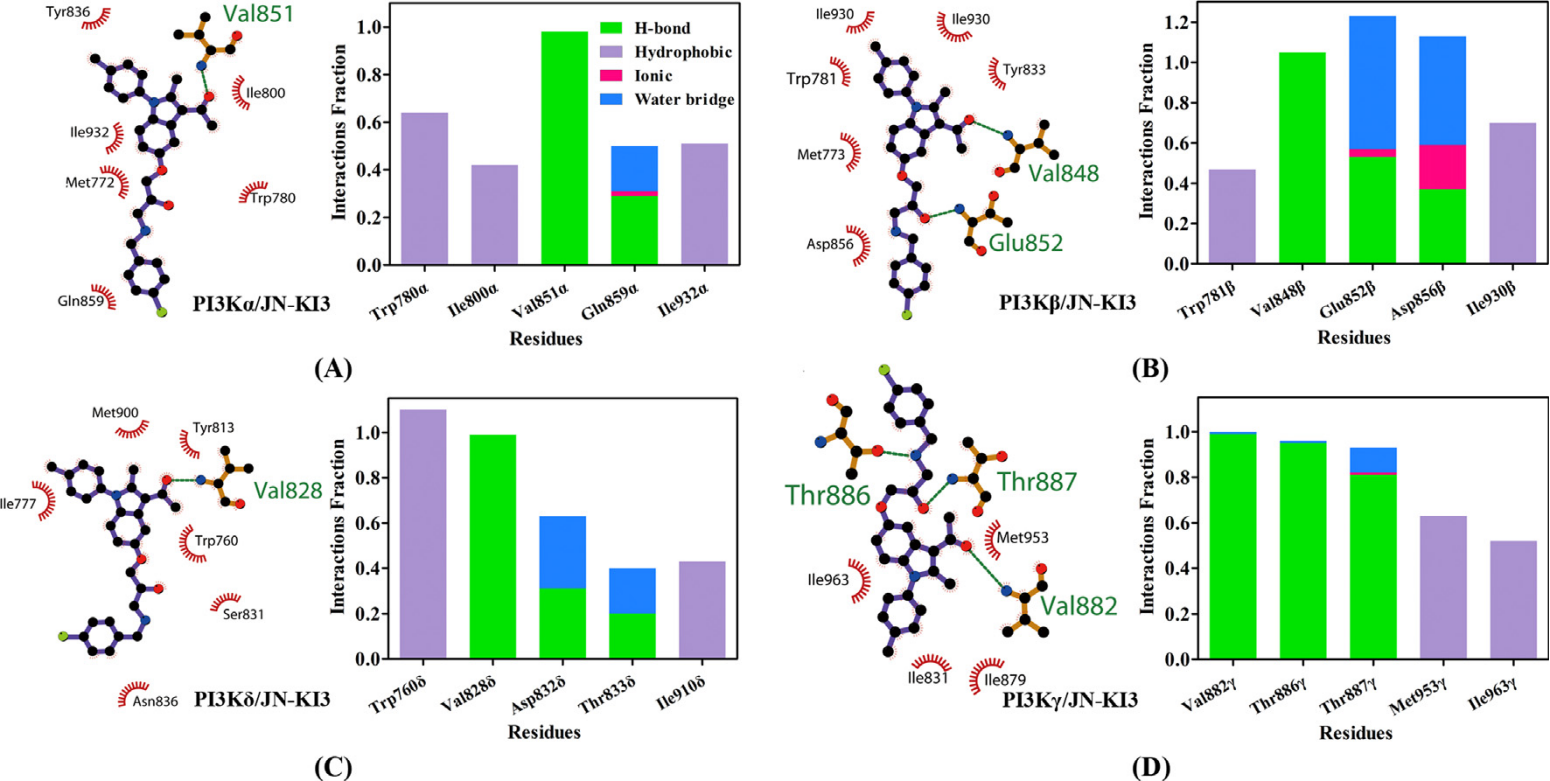
2D interaction patterns and protein–ligand occupancy histogram of **(A)** PI3Kα/JN-KI3; **(B)** PI3Kβ/JN-KI3; **(C)** PI3Kδ/JN-KI3; **(D)** PI3Kγ/JN-KI3 complexes.

To further uncover the interaction modes between JN-KI3 and PI3Ks in a dynamic process, the dominant interactions, including H-bond, hydrophobic, ionic interactions (Ionic) and hydrogen bonds through water molecular bridge (Water Bridge), between key residues of each PI3K isoform and JN-KI3 were elucidated. As shown in Fig. 5, the carbonyl group of JN-KI3 formed an H-bond with Val residue in all four PI3K isoforms, namely, Val851α, Val848β, Val828δ, and Val882γ. After a sequence comparison and structural alignment, it suggested that these Val residues were conserved and located at the similar position at the hinge region within the ATP-binding pocket in all four isoforms. The hinge region is one of the four well-depicted regions in the ATP-binding pocket playing a significant role in determining the binding affinities between PI3Ks and small molecular inhibitors. As for PI3Kα/JN-KI3, there were hydrophobic interactions between Trp780/Ile800/Ile932 and JN-KI3 (Fig. 5**A**). As for PI3Kβ/JN-KI3, Glu852 and Asp856 formed Water Bridge interactions with the free hydroxyl group and the secondary amino group of JN-KI3, respectively. Moreover, Ile930 formed a hydrophobic interaction with JN-KI3 (Fig. 5**B**). As for PI3Kδ/JN-KI3, Trp760 formed a Pi-Pi interaction with the indole ring of JN-KI3, which almost dominated the nonpolar contribution in PI3Kδ. In addition, there was a Water Bridge between Asp832 and JN-KI3 (Fig. 5**C**). As for PI3Kγ/JN-KI3, except for Val882, Thr887 formed an H-bond with the free hydroxyl group of JN-KI3, and Thr886 formed another H-bond with the free amino group of JN-KI3. As far as we know, these characteristic H-bonds are less common compared to most of the reported PI3K inhibitors. Besides, there were two strong hydrophobic interactions between Met953/Ile963 and JN-KI3 (Fig. 5**D**). Thus, the extra H-bonds and the stronger hydrophobic interactions between JN-KI3 and PI3Kγ were deemed as the critical reason for the PI3Kγ selective inhibition of JN-KI3.

**Fig. 6:**
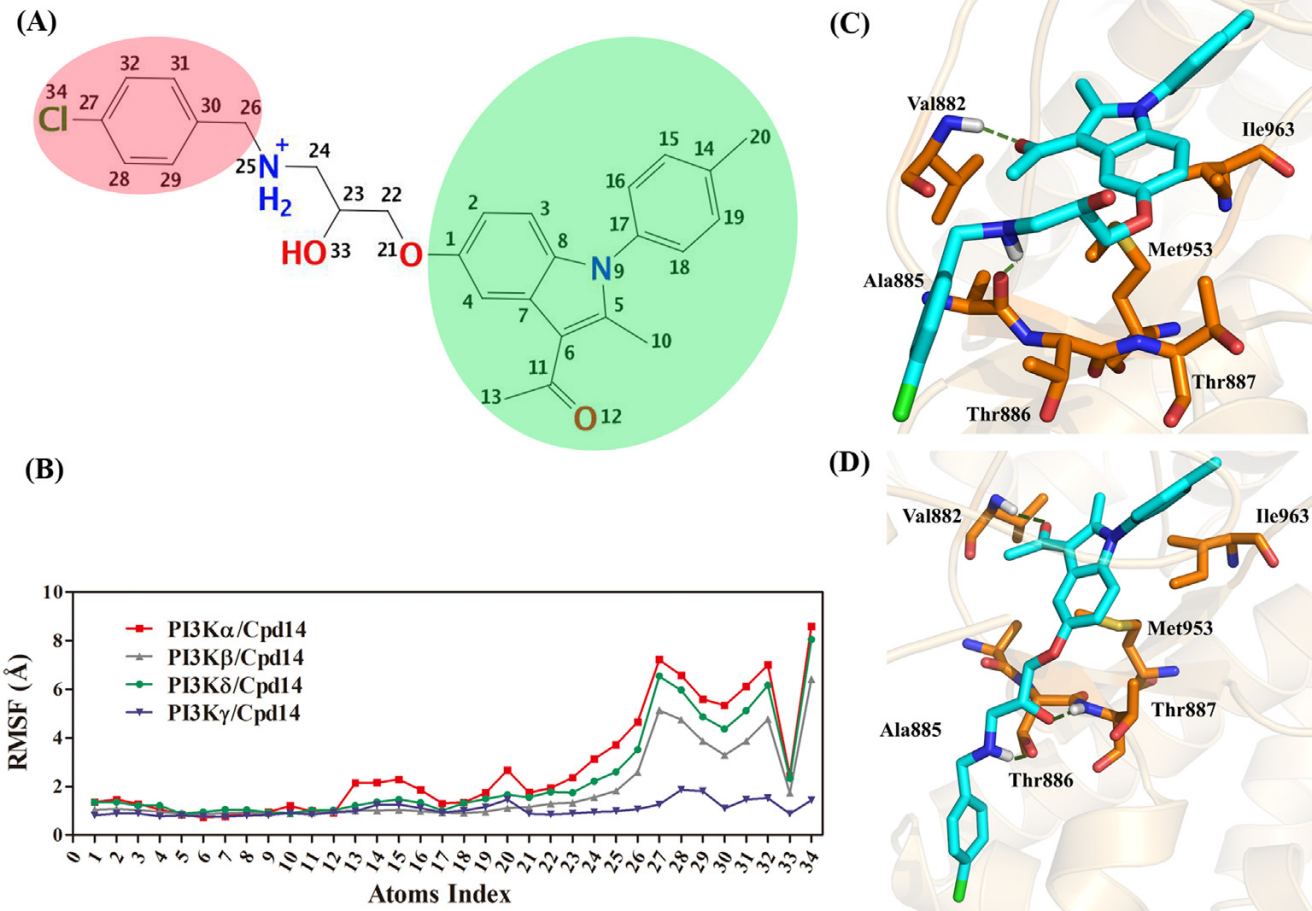
**(A)** the 2D-structure labeled with atomic numbers; **(B)** the ligand RMSF value of JN-KI3; **(C)** the 3D-interaction diagrams between JN-KI3 and PI3Kγ before MD simulation (H-bond colored in green); **(D)** the 3D-interaction diagrams between JN-KI3 and PI3Kγ after MD simulation (H-bond colored in green).

Finally, the RMSF value of each atom of JN-KI3 was calculated, the atoms forming potent interactions with PI3Ks would be imped to fluctuate and thus show extremely low values. As a whole, JN-KI3 exhibited less fluctuate in PI3Kγ system (Fig. 6**B)**, and it indicated JN-KI3 preferred to bind to PI3Kγ. Specifically, the carbonyl group (atom index: 11 and 12), the indole ring (atom index: 1–9), P-methylphenyl at 9 position (atom index: 14–20) were all bind to PI3Ks with almost same interacted effect (green colored in Fig. 6**A**). Comparing the binding poses before and after the MD simulation, these groups show little fluctuation in the PI3Kγ binding region (Fig. S6). The H-bond was always maintained between Val882 and the carbonyl group, while Met953 and Ile963 still form strong hydrophobic interactions with JN-KI3 (Fig. 6**C & D)**. Therefore, this motif of JN-KI3 is vital for the satisfactory binding affinity to PI3Kγ. However, the motif colored in red of JN-KI3 presented weak interactions with class IA PI3K isoforms, especially the chlorine phenyl (atom index: 26–32 and 34). Interestingly, the RMSF values of these atoms in PI3Kγ/JN-KI3 were far lower than the values in other systems. On the other hand, the H-bond between JN-KI3 and Ala885 lost after MD simulation (Fig. 6**C)**, the motif including chlorine phenyl (atom index: 21–34) had significant ligand torsions (Fig. S6**C**), that made the motif prefer to bias Thr886 and Thr887, and lastly formed two strong H-bonds with these two residues (Fig. 6**D).** Thus, all these results are supposed to be responsible for the PI3Kγ selective inhibition of JN-KI3.

## Conclusion

In the present study, a machine learning-based VS with multiple protein structures was developed to screen against a large chemical library. This integrated VS model exhibits more precision prediction through mimicking the induced-fit effect of drug target than conventional VS, and the sequential manner during this protocol could be more appropriate to balance the efficiency and precision of VS. The developed NBC model integrating ten PI3Kγ proteins showed a satisfactory prediction power against PI3Kγ inhibitors with the AUC value as high as 0.906, indicating that the VS with multiple PI3Kγ structures would get much better performance and higher enrichment rate than that based on any single PI3Kγ structure. After the evaluation of drug-likeness with the Lipinski and Veber rules and REOS, 49 VS-hits interacting with the “hot residues” within the ATP binding pocket of PI3Kγ were screened out, and then, all compounds were submitted to a series of bio-assay studies. Firstly, the PI3Kγ inhibitory activities of these hits were preliminarily validated by the cell-free assay, and four (4 in 49) compounds show over 50% inhibitory percent at a concentration of 10 μM. Among them, JN-KI3 exhibits the most potent selective PI3Kγ inhibitory bioactivity, the IC_50_ against PI3Kγ is 3,873 nM. And, even more crucially, JN-KI3 shows a significant specificity to PI3Kγ, while there is no inhibitory effect on Class IA PI3Ks even at concentrations higher than 20,000 nM. It suggests that the novel integrated VS strongly manifests the robustness to find new PI3Kγ inhibitors. Meanwhile, the *in silico* PAINS and ADMET assessments indicated that JN-KI3 contains appropriate drug-like properties. Subsequently, dozens of common human cancer cell lines were treated with JN-KI3, respectively, and it is interesting that JN-KI3 prefers to inhibit the proliferation of hematologic cancer cell lines, the inhibition effect on some hematologic cancer cells is even stronger than IPI-145, which is consistent with the main distribution and expression of PI3Kγ in the hematopoietic systems. Therefore, PI3Kγ would be a suitable therapeutic target for hematologic cancers. Immunoblotting analysis revealed that JN-KI3 would effectively inhibit PI3K/AKT signaling with concentration- and time-dependent manners in hematologic cancer cells even at a low concentration. The further studies at the cellular models show that JN-KI3 could significantly induce apoptosis of hematologic cancer cells. The potent selective inhibition on PI3Kγ of JN-KI3 resulted in the significant cleavage of two apoptosis biomarkers, PARP and caspase-3, and finally led to distinct apoptosis of the cell lines with dose- and time-dependent manners. Lastly, the selective inhibition mechanism of JN-KI3 against PI3Kγ was uncovered by a theoretical study combining molecular docking, MD simulation, and free energy calculations. The results reveal that JN-KI3 contains the highest binding free energy against PI3Kγ than Class IA isoforms, which is in good agreement with the experimental bioactivities. In addition, some key residues influencing γ isoform-selective inhibition of JN-KI3 were highlighted, such as Val882, Thr886, Thr887, Met953, and Ile963, these key interactions are less common compared to most of the reported PI3Kγ inhibitors, indicating that JN-KI3 has novel structural characteristics as a selective PI3Kγ inhibitor. On the other hand, some structural features of JN-KI3 critical to the γ-isoform preferred binding affinity were outstood, the chlorine phenyl of JN-KI3 formed stronger interactions with PI3Kγ than Class IA isoforms, which makes a favorable contribution for specific inhibition on PI3Kγ. All results could provide some significant guidance for the further rational design of JN-KI3.

## Author Contributions

ZJ, LH, HG, JJ developed the study concept and design. LK performed the modeling studies and the bio-assay evaluation. ZJ, LK, ZX carried out the data analysis. XL provided software. ZJ, LK, CY drafted the manuscript, CY, LH, HG, JJ approved the manuscript.

## Declaration of Competing Interest

The authors declare that they have no known competing financial interests or personal relationships that could have appeared to influence the work reported in this paper.
