# Supplementary material for: Discovery of novel selective PI3Kγ inhibitors through combining machine learning-based virtual screening with multiple protein structures and bio-evaluation
Source: J Adv Res. 2021 Apr 20;36:1–13. doi: 10.1016/j.jare.2021.04.007 (PMC8800018; doi:10.1016/j.jare.2021.04.007)
Supplement: Supplementary data 1 [file mmc1.docx]

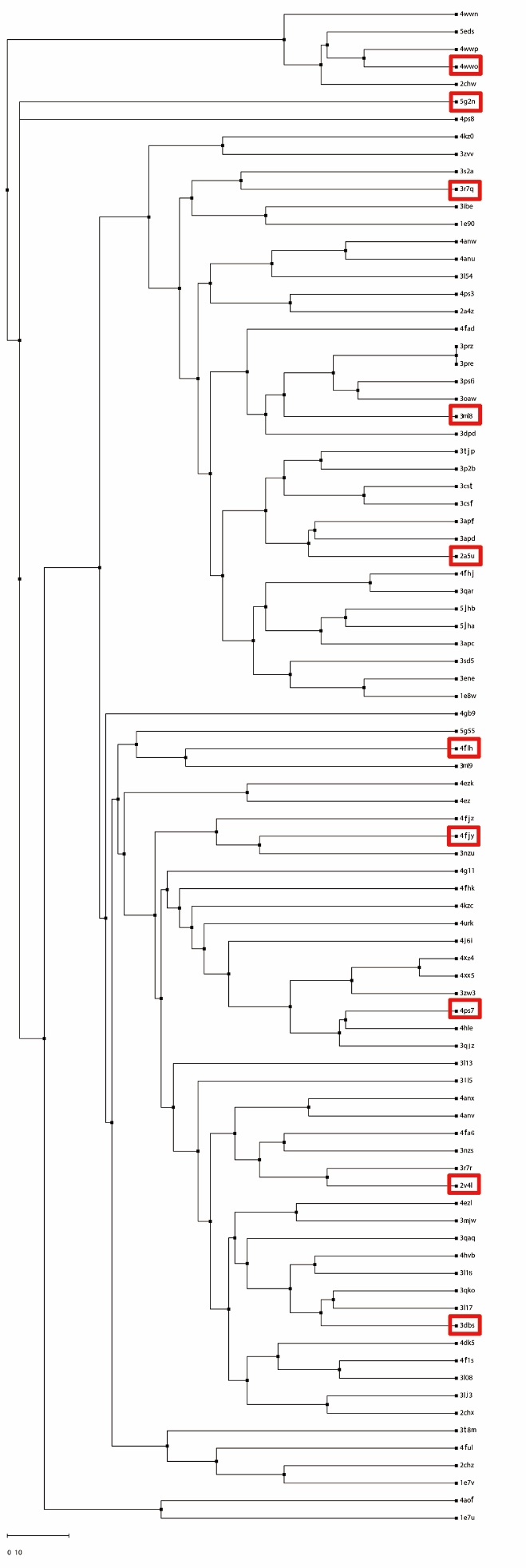


**Fig. S1**. The phylogenetic tree based on the calculated RMSD values of 87 PI3Kγ cryptographic structures, the ten selected complexes are highlighted in red


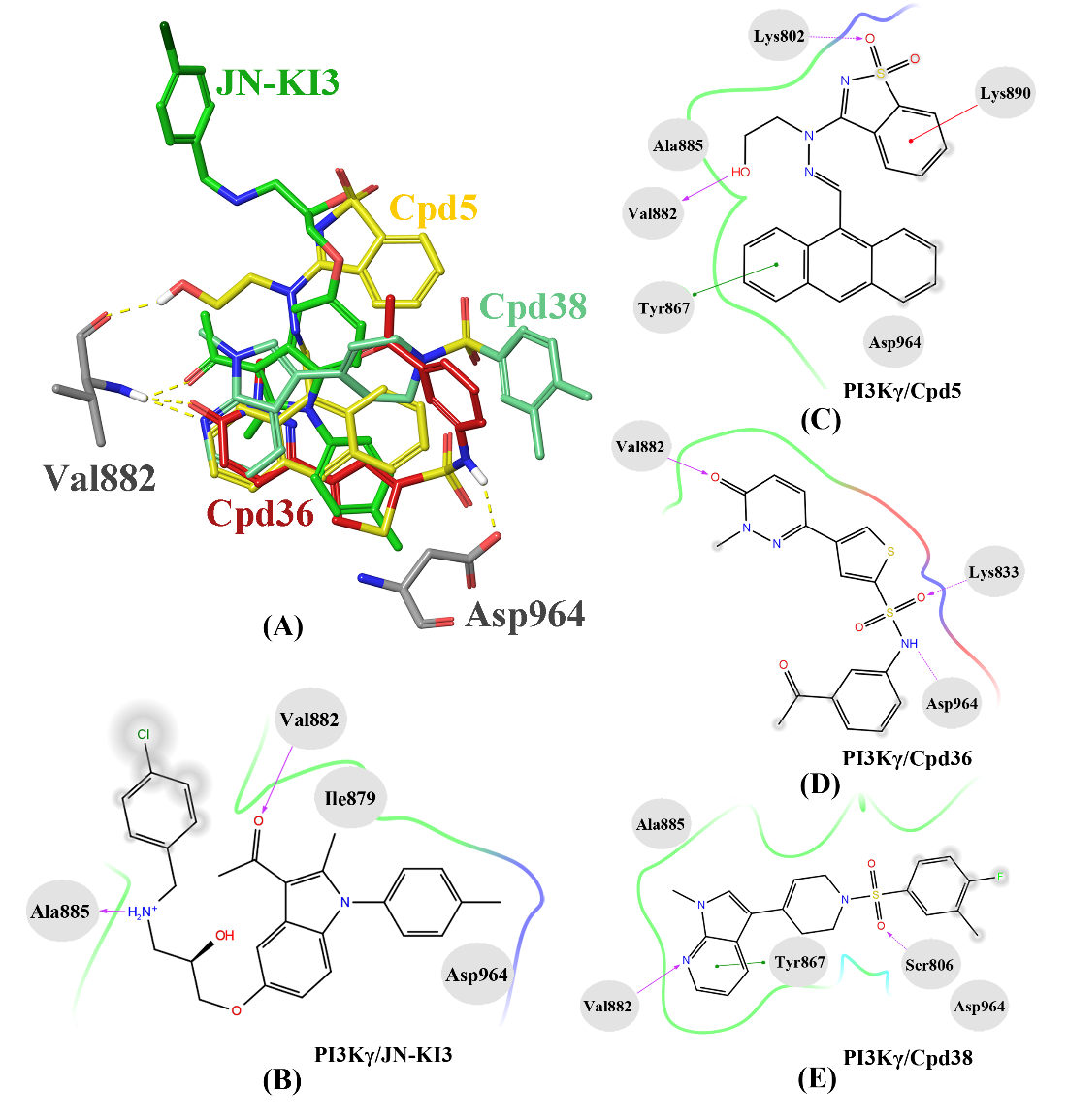


**Fig. S2.** **(A)** The alignment of Cpd5/JN-KI3 (Cpd14) /Cpd36/Cpd38 (H-bond colored in yellow); the docking poses of **(B)** PI3Kγ/JN-KI3 (Cpd14), **(C)** PI3Kγ/Cpd5, **(D)** PI3Kγ/Cpd36, and **(E)** PI3Kγ/Cpd38.


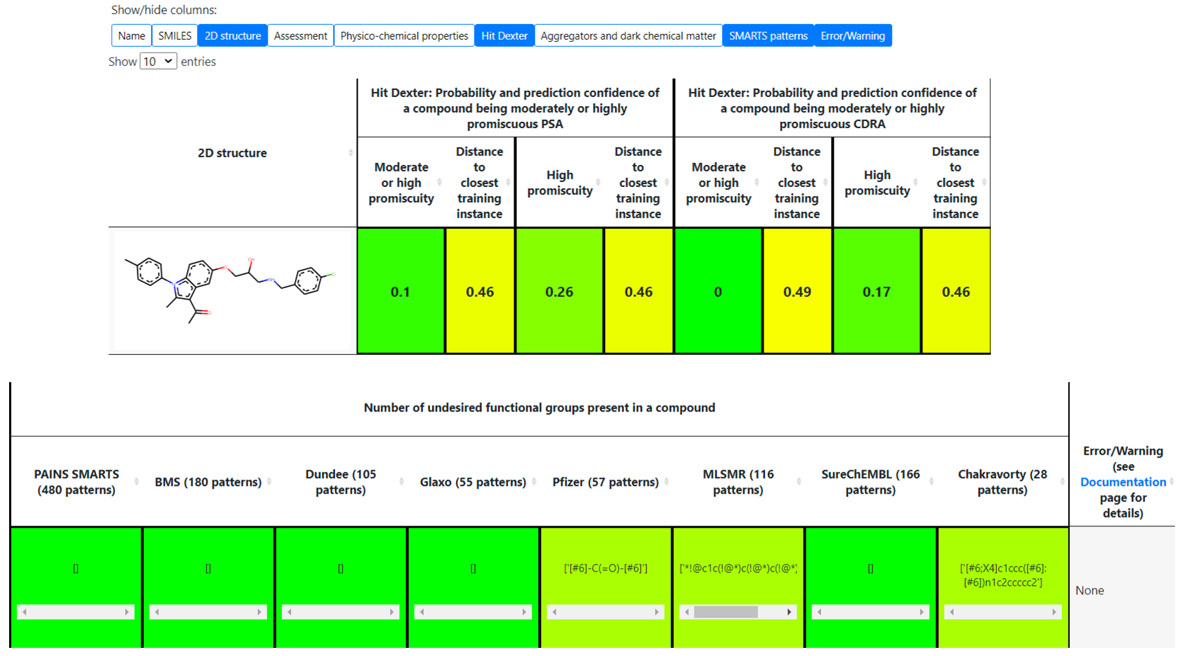


**Fig. S3.** The promiscuity analysis results of JN-KI3 using Hit Dexter 2.0.


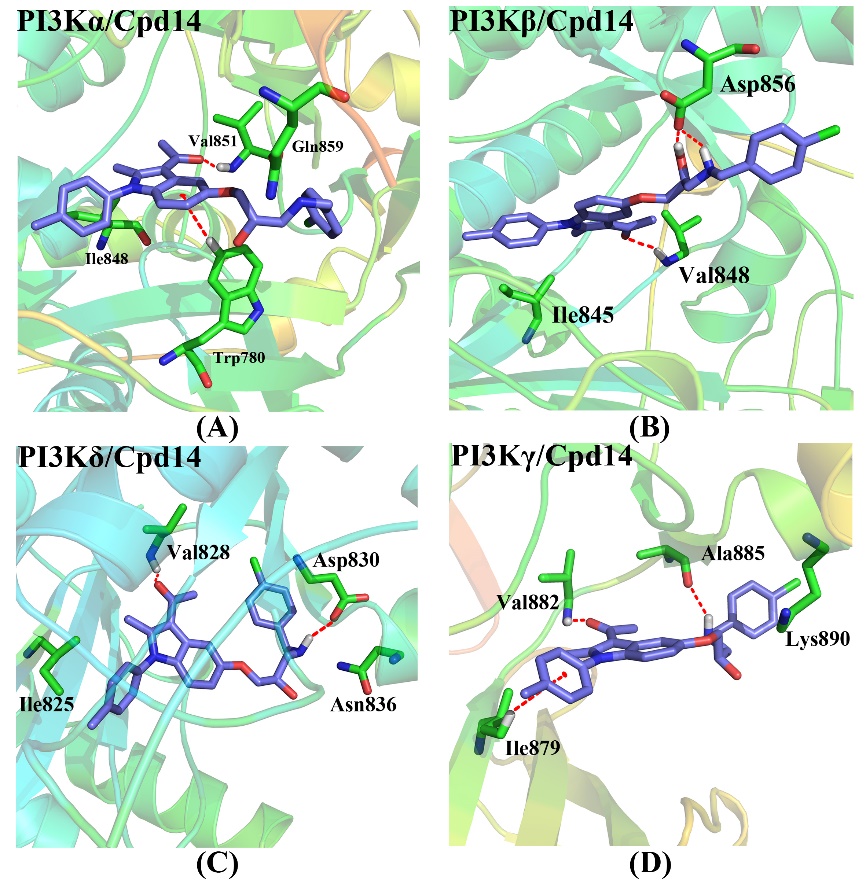


**Fig. S4.** The 3D-interaction diagrams between JN-KI3 and **(A)** PI3Kα; **(B)** PI3Kβ; **(C)** PI3Kδ; **(D)** PI3Kγ. The protein receptors are shown in a colored cartoon mode; the important amino acid residues are shown in a green stick model; JN-KI3 is shown in a royal blue stick model; hydrogen bonds are represented by red dashed lines.


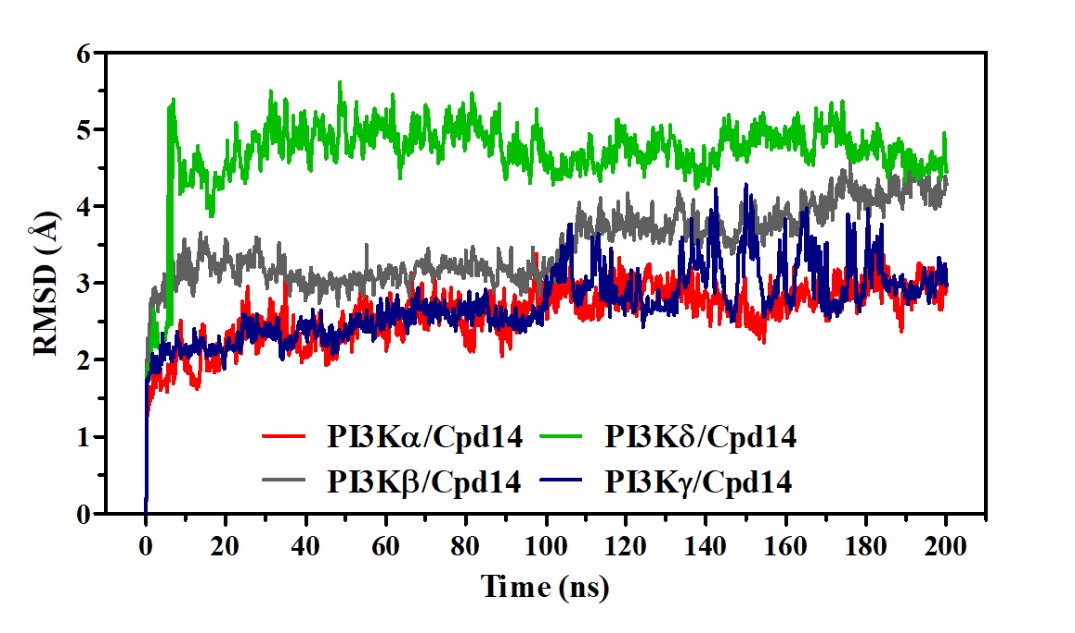


**Fig. S5.** The root-mean-square deviation (RMSDs) of the backbone atoms of PI3Ks/ JN-KI3 complexes.


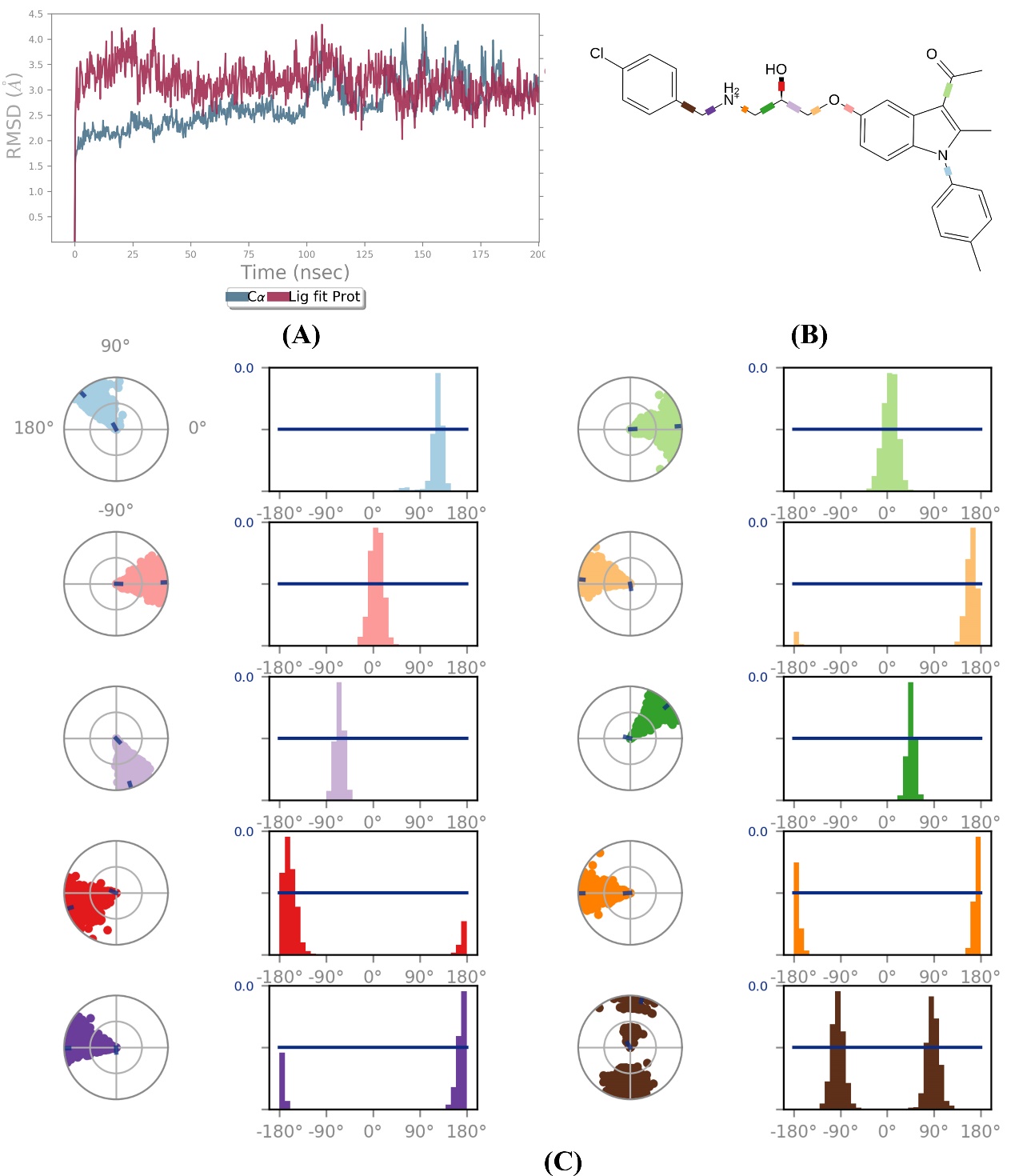


**Fig. S6.** **(A)** The root-mean-square deviation (RMSDs) of the backbone atoms of PI3Kγ and JN-KI3; **(B)** the rotatable bonds of JN-KI3; **(C)** the torsions plot of the rotatable bonds.

**Table S1.** The collected crystal structure of 87 PI3Kγ proteins for VS model.

| Number | PDB ID | Organisms | Reference |
| --- | --- | --- | --- |
| 1 | 1E90 | Sus scrofa | [1] |
| 2 | 1E7U | Sus scrofa | [1] |
| 3 | 1E7V | Sus scrofa | [1] |
| 4 | 1E8W | Sus scrofa | [1] |
| 5 | 1E8Z | Homo sapiens | [1] |
| 6 | 2A4Z | Homo sapiens | [2] |
| 7 | 2A5U | Homo sapiens | [2] |
| 8 | 2CHW | Homo sapiens | [3] |
| 9 | 2CHX | Homo sapiens | [3] |
| 10 | 2CHZ | Homo sapiens | [3] |
| 11 | 2V4L | Homo sapiens | [4] |
| 12 | 3ENE | Homo sapiens | [4] |
| 13 | 3LBE | Homo sapiens | [5] |
| 14 | 3APC | Homo sapiens | [6] |
| 15 | 3APD | Homo sapiens | [6] |
| 16 | 3APF | Homo sapiens | [6] |
| 17 | 3CSF | Homo sapiens | [7] |
| 18 | 3CST | Homo sapiens | [7] |
| 19 | 3DBS | Homo sapiens | [8] |
| 20 | 3NZS | Homo sapiens | [9] |
| 21 | 3NZU | Homo sapiens | [9] |
| 22 | 3L08 | Homo sapiens | [10] |
| 23 | 3L54 | Homo sapiens | [10] |
| 24 | 3L13 | Homo sapiens | [11] |
| 25 | 3L16 | Homo sapiens | [11] |
| 26 | 3L17 | Homo sapiens | [11] |
| 27 | 3LJ3 | Homo sapiens | [12] |
| 28 | 3MJW | Homo sapiens | [13] |
| 29 | 3ML8 | Homo sapiens | [14] |
| 30 | 3ML9 | Homo sapiens | [14] |
| 31 | 4HVB | Homo sapiens | [14] |
| 32 | 3OAW | Homo sapiens | [15] |
| 33 | 3P2B | Homo sapiens | [16] |
| 34 | 3PRE | Homo sapiens | [17] |
| 35 | 3PRZ | Homo sapiens | [17] |
| 36 | 3PS6 | Homo sapiens | [17] |
| 37 | 3QAQ | Homo sapiens | [18] |
| 38 | 3QAR | Homo sapiens | [18] |
| 39 | 3QJZ | Homo sapiens | [19] |
| 40 | 3QK0 | Homo sapiens | [19] |
| 41 | 3DPD | Homo sapiens | [20] |
| 42 | 3R7Q | Homo sapiens | [21] |
| 43 | 3R7R | Homo sapiens | [21] |
| 44 | 3S2A | Homo sapiens | [22] |
| 45 | 3SD5 | Homo sapiens | [23] |
| 46 | 3TL5 | Homo sapiens | [21] |
| 47 | 3ZVV | Homo sapiens | [24] |
| 48 | 3ZW3 | Homo sapiens | [24] |
| 49 | 4J6I | Homo sapiens | [25] |
| 50 | 4ANU | Homo sapiens | [26] |
| 51 | 4ANW | Homo sapiens | [26] |
| 52 | 4ANX | Homo sapiens | [26] |
| 53 | 4AOF | Homo sapiens | [27] |
| 54 | 4DK5 | Homo sapiens | [28] |
| 55 | 4EZJ | Homo sapiens | [28] |
| 56 | 4EZK | Homo sapiens | [28] |
| 57 | 4EZL | Homo sapiens | [28] |
| 58 | 4F1S | Homo sapiens | [29] |
| 59 | 4FA6 | Homo sapiens | [30] |
| 60 | 4FAD | Homo sapiens | [30] |
| 61 | 4FHJ | Homo sapiens | [31] |
| 62 | 4FHK | Homo sapiens | [31] |
| 63 | 4FJY | Homo sapiens | [32] |
| 64 | 4FJZ | Homo sapiens | [32] |
| 65 | 4FLH | Homo sapiens | [33] |
| 66 | 4FUL | Homo sapiens | [34] |
| 67 | 4G11 | Homo sapiens | [35] |
| 68 | 4GB9 | Homo sapiens | [36] |
| 69 | 4HLE | Homo sapiens | [37] |
| 70 | 4KZC | Homo sapiens | [38] |
| 71 | 4KZO | Homo sapiens | [38] |
| 72 | 4PS3 | Homo sapiens | [39] |
| 73 | 4PS7 | Homo sapiens | [39] |
| 74 | 4PS8 | Homo sapiens | [39] |
| 75 | 4URK | Homo sapiens | [40] |
| 76 | 4WWN | Homo sapiens | [41] |
| 77 | 4WWO | Homo sapiens | [41] |
| 78 | 4WWP | Homo sapiens | [41] |
| 79 | 5EDS | Homo sapiens | [42] |
| 80 | 5G2N | Homo sapiens | [43] |
| 81 | 5G55 | Homo sapiens | [44] |
| 82 | 5JHA | Homo sapiens | [45] |
| 83 | 5JHB | Homo sapiens | [45] |
| 84 | 3T8M | Homo sapiens | To be published |
| 85 | 3TJP | Homo sapiens | To be published |
| 86 | 4XX5 | Homo sapiens | To be published |
| 87 | 4XZ4 | Homo sapiens | To be published |

**Table S2.** The 2D-structures, docking scores and the existing interactions of Cpd5, JN-KI3 (Cpd14), Cpd36 and Cpd38 with PI3Kγ

| Compounds | Structure | Docking Score  (Kcal/mol) | Interactions  with PI3Kγ |
| --- | --- | --- | --- |
| Cpd5 | 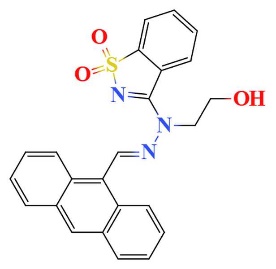 | -7.425 | Val882, Tyr867, Lys890, Lys802 |
| JN-KI3 | 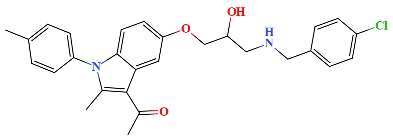 | -7.841 | Val882, Ala885 |
| Cpd36 | 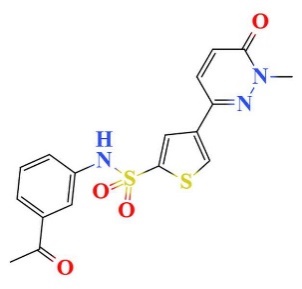 | -7.518 | Val882, Lys833, Asp964 |
| Cpd38 | 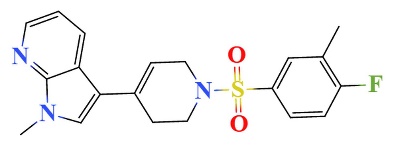 | -7.488 | Val882, Tyr867, Ser806 |

**References**

[1] E.H. Walker, M.E. Pacold, O. Perisic, L. Stephens, P.T. Hawkins, M.P. Wymann, R.L. Williams, Structural determinants of phosphoinositide 3-kinase inhibition by wortmannin, LY294002, quercetin, myricetin, and staurosporine, Mol. Cell 6 (2000) 909-919.

[2] M. Camps, T. Ruckle, H. Ji, V. Ardissone, F. Rintelen, J. Shaw, C. Ferrandi, C. Chabert, C. Gillieron, B. Francon, T. Martin, D. Gretener, D. Perrin, D. Leroy, P.A. Vitte, E. Hirsch, M.P. Wymann, R. Cirillo, M.K. Schwarz, C. Rommel, Blockade of PI3Kgamma suppresses joint inflammation and damage in mouse models of rheumatoid arthritis, Nat. Med. 11 (2005) 936-943.

[3] Z.A. Knight, B. Gonzalez, M.E. Feldman, E.R. Zunder, D.D. Goldenberg, O. Williams, R. Loewith, D. Stokoe, A. Balla, B. Toth, T. Balla, W.A. Weiss, R.L. Williams, K.M. Shokat, A pharmacological map of the PI3-K family defines a role for p110alpha in insulin signaling, Cell 125 (2006) 733-747.

[4] B. Apsel, J.A. Blair, B. Gonzalez, T.M. Nazif, M.E. Feldman, B. Aizenstein, R. Hoffman, R.L. Williams, K.M. Shokat, Z.A. Knight, Targeted polypharmacology: discovery of dual inhibitors of tyrosine and phosphoinositide kinases, Nat. Chem. Biol. 4 (2008) 691-699.

[5] A. Zask, J.C. Verheijen, K. Curran, J. Kaplan, D.J. Richard, P. Nowak, D.J. Malwitz, N. Brooijmans, J. Bard, K. Svenson, J. Lucas, L. Toral-Barza, W.G. Zhang, I. Hollander, J.J. Gibbons, R.T. Abraham, S. Ayral-Kaloustian, T.S. Mansour, K. Yu, ATP-competitive inhibitors of the mammalian target of rapamycin: design and synthesis of highly potent and selective pyrazolopyrimidines, J. Med. Chem. 52 (2009) 5013-5016.

[6] J. Ohwada, H. Ebiike, H. Kawada, M. Tsukazaki, M. Nakamura, T. Miyazaki, K. Morikami, K. Yoshinari, M. Yoshida, O. Kondoh, S. Kuramoto, K. Ogawa, Y. Aoki, N. Shimma, Discovery and biological activity of a novel class I PI3K inhibitor, CH5132799, Bioorg. Med. Chem. Lett. 21 (2011) 1767-1772.

[7] P. Xie, D.S. Williams, G.E. Atilla-Gokcumen, L. Milk, M. Xiao, K.S. Smalley, M. Herlyn, E. Meggers, R. Marmorstein, Structure-based design of an organoruthenium phosphatidyl-inositol-3-kinase inhibitor reveals a switch governing lipid kinase potency and selectivity, ACS Chem. Biol. 3 (2008) 305-316.

[8] A.J. Folkes, K. Ahmadi, W.K. Alderton, S. Alix, S.J. Baker, G. Box, I.S. Chuckowree, P.A. Clarke, P. Depledge, S.A. Eccles, L.S. Friedman, A. Hayes, T.C. Hancox, A. Kugendradas, L. Lensun, P. Moore, A.G. Olivero, J. Pang, S. Patel, G.H. Pergl-Wilson, F.I. Raynaud, A. Robson, N. Saghir, L. Salphati, S. Sohal, M.H. Ultsch, M. Valenti, H.J. Wallweber, N.C. Wan, C. Wiesmann, P. Workman, A. Zhyvoloup, M.J. Zvelebil, S.J. Shuttleworth, The identification of 2-(1H-indazol-4-yl)-6-(4-methanesulfonyl-piperazin-1-ylmethyl)-4-morpholin-4-yl-t hieno[3,2-d]pyrimidine (GDC-0941) as a potent, selective, orally bioavailable inhibitor of class I PI3 kinase for the treatment of cancer, J. Med. Chem. 51 (2008) 5522-5532.

[9] S.T. Staben, T.P. Heffron, D.P. Sutherlin, S.R. Bhat, G.M. Castanedo, I.S. Chuckowree, J. Dotson, A.J. Folkes, L.S. Friedman, L. Lee, J. Lesnick, C. Lewis, J.M. Murray, J. Nonomiya, A.G. Olivero, E. Plise, J. Pang, W.W. Prior, L. Salphati, L. Rouge, D. Sampath, V. Tsui, N.C. Wan, S. Wang, C. Weismann, P. Wu, B.Y. Zhu, Structure-based optimization of pyrazolo-pyrimidine and -pyridine inhibitors of PI3-kinase, Bioorg. Med. Chem. Lett. 20 (2010) 6048-6051.

[10] S.D. Knight, N.D. Adams, J.L. Burgess, A.M. Chaudhari, M.G. Darcy, C.A. Donatelli, J.I. Luengo, K.A. Newlander, C.A. Parrish, L.H. Ridgers, M.A. Sarpong, S.J. Schmidt, G.S. Van Aller, J.D. Carson, M.A. Diamond, P.A. Elkins, C.M. Gardiner, E. Garver, S.A. Gilbert, R.R. Gontarek, J.R. Jackson, K.L. Kershner, L. Luo, K. Raha, C.S. Sherk, C.M. Sung, D. Sutton, P.J. Tummino, R.J. Wegrzyn, K.R. Auger, D. Dhanak, Discovery of GSK2126458, a Highly Potent Inhibitor of PI3K and the Mammalian Target of Rapamycin, ACS Med. Chem. Lett. 1 (2010) 39-43.

[11] D.P. Sutherlin, D. Sampath, M. Berry, G. Castanedo, Z. Chang, I. Chuckowree, J. Dotson, A. Folkes, L. Friedman, R. Goldsmith, T. Heffron, L. Lee, J. Lesnick, C. Lewis, S. Mathieu, J. Nonomiya, A. Olivero, J. Pang, W.W. Prior, L. Salphati, S. Sideris, Q. Tian, V. Tsui, N.C. Wan, S. Wang, C. Wiesmann, S. Wong, B.Y. Zhu, Discovery of (thienopyrimidin-2-yl)aminopyrimidines as potent, selective, and orally available pan-PI3-kinase and dual pan-PI3-kinase/mTOR inhibitors for the treatment of cancer, J. Med. Chem. 53 (2010) 1086-1097.

[12] H.R. Tsou, G. MacEwan, G. Birnberg, G. Grosu, M.G. Bursavich, J. Bard, N. Brooijmans, L. Toral-Barza, I. Hollander, T.S. Mansour, S. Ayral-Kaloustian, K. Yu, Discovery and optimization of 2-(4-substituted-pyrrolo[2,3-b]pyridin-3-yl)methylene-4-hydroxybenzofuran-3(2H)-o nes as potent and selective ATP-competitive inhibitors of the mammalian target of rapamycin (mTOR), Bioorg. Med. Chem. Lett. 20 (2010) 2321-2325.

[13] N. Zhang, S. Ayral-Kaloustian, J.T. Anderson, T. Nguyen, S. Das, A.M. Venkatesan, N. Brooijmans, J. Lucas, K. Yu, I. Hollander, R. Mallon, 5-ureidobenzofuranone indoles as potent and efficacious inhibitors of PI3 kinase-alpha and mTOR for the treatment of breast cancer, Bioorg. Med. Chem. Lett. 20 (2010) 3526-3529.

[14] H. Cheng, C. Li, S. Bailey, S.M. Baxi, L. Goulet, L. Guo, J. Hoffman, Y. Jiang, T.O. Johnson, T.W. Johnson, D.R. Knighton, J. Li, K.K. Liu, Z. Liu, M.A. Marx, M. Walls, P.A. Wells, M.J. Yin, J. Zhu, M. Zientek, Discovery of the Highly Potent PI3K/mTOR Dual Inhibitor PF-04979064 through Structure-Based Drug Design, ACS Med. Chem. Lett. 4 (2013) 91-97.

[15] K.K. Liu, S. Bagrodia, S. Bailey, H. Cheng, H. Chen, L. Gao, S. Greasley, J.E. Hoffman, Q. Hu, T.O. Johnson, D. Knighton, Z. Liu, M.A. Marx, M.D. Nambu, S. Ninkovic, B. Pascual, K. Rafidi, C.M. Rodgers, G.L. Smith, S. Sun, H. Wang, A. Yang, J. Yuan, A. Zou, 4-methylpteridinones as orally active and selective PI3K/mTOR dual inhibitors, Bioorg. Med. Chem. Lett. 20 (2010) 6096-6099.

[16] S. Pecchi, P.A. Renhowe, C. Taylor, S. Kaufman, H. Merritt, M. Wiesmann, K.R. Shoemaker, M.S. Knapp, E. Ornelas, T.F. Hendrickson, W. Fantl, C.F. Voliva, Identification and structure-activity relationship of 2-morpholino 6-(3-hydroxyphenyl) pyrimidines, a class of potent and selective PI3 kinase inhibitors, Bioorg. Med. Chem. Lett. 20 (2010) 6895-6898.

[17] K.K. Liu, X. Huang, S. Bagrodia, J.H. Chen, S. Greasley, H. Cheng, S. Sun, D. Knighton, C. Rodgers, K. Rafidi, A. Zou, J. Xiao, S. Yan, Quinazolines with intra-molecular hydrogen bonding scaffold (iMHBS) as PI3K/mTOR dual inhibitors, Bioorg. Med. Chem. Lett. 21 (2011) 1270-1274.

[18] E.A. Peterson, P.S. Andrews, X. Be, A.A. Boezio, T.L. Bush, A.C. Cheng, J.R. Coats, A.E. Colletti, K.W. Copeland, M. DuPont, R. Graceffa, B. Grubinska, J.C. Harmange, J.L. Kim, E.L. Mullady, P. Olivieri, L.B. Schenkel, M.K. Stanton, Y. Teffera, D.A. Whittington, T. Cai, D.S. La, Discovery of triazine-benzimidazoles as selective inhibitors of mTOR, Bioorg. Med. Chem. Lett. 21 (2011) 2064-2070.

[19] N.D. D'Angelo, T.S. Kim, K. Andrews, S.K. Booker, S. Caenepeel, K. Chen, D. D'Amico, D. Freeman, J. Jiang, L. Liu, J.D. McCarter, T. San Miguel, E.L. Mullady, M. Schrag, R. Subramanian, J. Tang, R.C. Wahl, L. Wang, D.A. Whittington, T. Wu, N. Xi, Y. Xu, P. Yakowec, K. Yang, L.P. Zalameda, N. Zhang, P. Hughes, M.H. Norman, Discovery and optimization of a series of benzothiazole phosphoinositide 3-kinase (PI3K)/mammalian target of rapamycin (mTOR) dual inhibitors, J. Med. Chem. 54 (2011) 1789-1811.

[20] B. Perry, R. Alexander, G. Bennett, G. Buckley, T. Ceska, T. Crabbe, V. Dale, L. Gowers, H. Horsley, L. James, K. Jenkins, K. Crepy, C. Kulisa, H. Lightfoot, C. Lock, S. Mack, T. Morgan, A.L. Nicolas, W. Pitt, V. Sabin, S. Wright, Achieving multi-isoform PI3K inhibition in a series of substituted 3,4-dihydro-2H-benzo[1,4]oxazines, Bioorg. Med. Chem. Lett. 18 (2008) 4700-4704.

[21] S.T. Staben, M. Siu, R. Goldsmith, A.G. Olivero, S. Do, D.J. Burdick, T.P. Heffron, J. Dotson, D.P. Sutherlin, B.-Y. Zhu, V. Tsui, H. Le, L. Lee, J. Lesnick, C. Lewis, J.M. Murray, J. Nonomiya, J. Pang, W.W. Prior, L. Salphati, L. Rouge, D. Sampath, S. Sideris, C. Wiesmann, P. Wu, Structure-based design of thienobenzoxepin inhibitors of PI3-kinase, Bioorg. Med. Chem. Lett. 21 (2011) 4054-4058.

[22] N. Nishimura, A. Siegmund, L. Liu, K. Yang, M.C. Bryan, K.L. Andrews, Y. Bo, S.K. Booker, S. Caenepeel, D. Freeman, H. Liao, J. McCarter, E.L. Mullady, T. San Miguel, R. Subramanian, N. Tamayo, L. Wang, D.A. Whittington, L. Zalameda, N. Zhang, P.E. Hughes, M.H. Norman, Phospshoinositide 3-kinase (PI3K)/mammalian target of rapamycin (mTOR) dual inhibitors: discovery and structure-activity relationships of a series of quinoline and quinoxaline derivatives, J. Med. Chem. 54 (2011) 4735-4751.

[23] S.-M. Maira, S. Pecchi, A. Huang, M. Burger, M. Knapp, D. Sterker, C. Schnell, D. Guthy, T. Nagel, M. Wiesmann, S. Brachmann, C. Fritsch, M. Dorsch, P. Chène, K. Shoemaker, A. De Pover, D. Menezes, G. Martiny-Baron, D. Fabbro, C.J. Wilson, R. Schlegel, F. Hofmann, C. García-Echeverría, W.R. Sellers, C.F. Voliva, Identification and characterization of NVP-BKM120, an orally available pan-class I PI3-kinase inhibitor, Mol. Cancer Therap. 11 (2012) 317-328.

[24] S.J. Hughes, D.S. Millan, I.C. Kilty, R.A. Lewthwaite, J.P. Mathias, M.A. O'Reilly, A. Pannifer, A. Phelan, F. Stühmeier, D.A. Baldock, D.G. Brown, Fragment based discovery of a novel and selective PI3 kinase inhibitor, Bioorg. Med. Chem. Lett. 21 (2011) 6586-6590.

[25] S.T. Staben, C. Ndubaku, N. Blaquiere, M. Belvin, R.J. Bull, D. Dudley, K. Edgar, D. Gray, R. Heald, T.P. Heffron, G.E. Jones, M. Jones, A. Kolesnikov, L. Lee, J. Lesnick, C. Lewis, J. Murray, N.J. McLean, J. Nonomiya, A.G. Olivero, R. Ord, J. Pang, S. Price, W.W. Prior, L. Rouge, L. Salphati, D. Sampath, J. Wallin, L. Wang, B. Wei, C. Weismann, P. Wu, Discovery of thiazolobenzoxepin PI3-kinase inhibitors that spare the PI3-kinase β isoform, Bioorg. Med. Chem. Lett. 23 (2013) 2606-2613.

[26] J.W. Leahy, C.A. Buhr, H.W. Johnson, B.G. Kim, T. Baik, J. Cannoy, T.P. Forsyth, J.W. Jeong, M.S. Lee, S. Ma, K. Noson, L. Wang, M. Williams, J.M. Nuss, E. Brooks, P. Foster, L. Goon, N. Heald, C. Holst, C. Jaeger, S. Lam, J. Lougheed, L. Nguyen, A. Plonowski, J. Song, T. Stout, X. Wu, M.F. Yakes, P. Yu, W. Zhang, P. Lamb, O. Raeber, Discovery of a novel series of potent and orally bioavailable phosphoinositide 3-kinase gamma inhibitors, J. Med. Chem. 55 (2012) 5467-5482.

[27] G. Bergamini, K. Bell, S. Shimamura, T. Werner, A. Cansfield, K. Müller, J. Perrin, C. Rau, K. Ellard, C. Hopf, C. Doce, D. Leggate, R. Mangano, T. Mathieson, A. O'Mahony, I. Plavec, F. Rharbaoui, F. Reinhard, M.M. Savitski, N. Ramsden, E. Hirsch, G. Drewes, O. Rausch, M. Bantscheff, G. Neubauer, A selective inhibitor reveals PI3Kγ dependence of T(H)17 cell differentiation, Nat. Chem. Biol. 8 (2012) 576-582.

[28] D.P. Sutherlin, S. Baker, A. Bisconte, P.M. Blaney, A. Brown, B.K. Chan, D. Chantry, G. Castanedo, P. DePledge, P. Goldsmith, D.M. Goldstein, T. Hancox, J. Kaur, D. Knowles, R. Kondru, J. Lesnick, M.C. Lucas, C. Lewis, J. Murray, A.J. Nadin, J. Nonomiya, J. Pang, N. Pegg, S. Price, K. Reif, B.S. Safina, L. Salphati, S. Staben, E.M. Seward, S. Shuttleworth, S. Sohal, Z.K. Sweeney, M. Ultsch, B. Waszkowycz, B. Wei, Potent and selective inhibitors of PI3Kdelta: obtaining isoform selectivity from the affinity pocket and tryptophan shelf, Bioorg. Med. Chem. Lett. 22 (2012) 4296-4302.

[29] R.P. Wurz, L. Liu, K. Yang, N. Nishimura, Y. Bo, L.H. Pettus, S. Caenepeel, D.J. Freeman, J.D. McCarter, E.L. Mullady, T.S. Miguel, L. Wang, N. Zhang, K.L. Andrews, D.A. Whittington, J. Jiang, R. Subramanian, P.E. Hughes, M.H. Norman, Synthesis and structure-activity relationships of dual PI3K/mTOR inhibitors based on a 4-amino-6-methyl-1,3,5-triazine sulfonamide scaffold, Bioorg. Med. Chem. Lett. 22 (2012) 5714-5720.

[30] P.T. Le, H. Cheng, S. Ninkovic, M. Plewe, X. Huang, H. Wang, S. Bagrodia, S. Sun, D.R. Knighton, C.M. LaFleur Rogers, A. Pannifer, S. Greasley, D. Dalvie, E. Zhang, Design and synthesis of a novel pyrrolidinyl pyrido pyrimidinone derivative as a potent inhibitor of PI3Kα and mTOR, Bioorg. Med. Chem. Lett. 22 (2012) 5098-5103.

[31] E.A. Peterson, A.A. Boezio, P.S. Andrews, C.M. Boezio, T.L. Bush, A.C. Cheng, D. Choquette, J.R. Coats, A.E. Colletti, K.W. Copeland, M. DuPont, R. Graceffa, B. Grubinska, J.L. Kim, R.T. Lewis, J. Liu, E.L. Mullady, M.H. Potashman, K. Romero, P.L. Shaffer, M.K. Stanton, J.C. Stellwagen, Y. Teffera, S. Yi, T. Cai, D.S. La, Discovery and optimization of potent and selective imidazopyridine and imidazopyridazine mTOR inhibitors, Bioorg. Med. Chem. Lett. 22 (2012) 4967-4974.

[32] F. Gonzalez-Lopez de Turiso, Y. Shin, M. Brown, M. Cardozo, Y. Chen, D. Fong, X. Hao, X. He, K. Henne, Y.-L. Hu, M.G. Johnson, T. Kohn, J. Lohman, H.J. McBride, L.R. McGee, J.C. Medina, D. Metz, K. Miner, D. Mohn, V. Pattaropong, J. Seganish, J.L. Simard, S. Wannberg, D.A. Whittington, G. Yu, T.D. Cushing, Discovery and in vivo evaluation of dual PI3Kβ/δ inhibitors, J. Med. Chem. 55 (2012) 7667-7685.

[33] M.H. Norman, K.L. Andrews, Y.Y. Bo, S.K. Booker, S. Caenepeel, V.J. Cee, N.D. D'Angelo, D.J. Freeman, B.J. Herberich, F.-T. Hong, C.L.M. Jackson, J. Jiang, B.A. Lanman, L. Liu, J.D. McCarter, E.L. Mullady, N. Nishimura, L.H. Pettus, A.B. Reed, T.S. Miguel, A.L. Smith, M.M. Stec, S. Tadesse, A. Tasker, D. Aidasani, X. Zhu, R. Subramanian, N.A. Tamayo, L. Wang, D.A. Whittington, B. Wu, T. Wu, R.P. Wurz, K. Yang, L. Zalameda, N. Zhang, P.E. Hughes, Selective class I phosphoinositide 3-kinase inhibitors: optimization of a series of pyridyltriazines leading to the identification of a clinical candidate, AMG 511, J. Med. Chem. 55 (2012) 7796-7816.

[34] A. Gopalsamy, E.M. Bennett, M. Shi, W.-G. Zhang, J. Bard, K. Yu, Identification of pyrimidine derivatives as hSMG-1 inhibitors, Bioorg. Med. Chem. Lett. 22 (2012) 6636-6641.

[35] V. Certal, F. Halley, A. Virone-Oddos, F. Thompson, B. Filoche-Rommé, Y. El-Ahmad, J.-C. Carry, C. Delorme, A. Karlsson, P.-Y. Abecassis, L. Vincent, H. Bonnevaux, J.-P. Nicolas, R. Morales, N. Michot, I. Vade, A. Louboutin, S. Perron, G. Doerflinger, B. Tric, S. Monget, C. Lengauer, L. Schio, Preparation and optimization of new 4-(morpholin-4-yl)-(6-oxo-1,6-dihydropyrimidin-2-yl)amide derivatives as PI3Kβ inhibitors, Bioorg. Med. Chem. Lett. 22 (2012) 6381-6384.

[36] J.M. Murray, Z.K. Sweeney, B.K. Chan, M. Balazs, E. Bradley, G. Castanedo, C. Chabot, D. Chantry, M. Flagella, D.M. Goldstein, R. Kondru, J. Lesnick, J. Li, M.C. Lucas, J. Nonomiya, J. Pang, S. Price, L. Salphati, B. Safina, P.P.A. Savy, E.M. Seward, M. Ultsch, D.P. Sutherlin, Potent and highly selective benzimidazole inhibitors of PI3-kinase delta, J. Med. Chem. 55 (2012) 7686-7695.

[37] S.T. Staben, N. Blaquiere, V. Tsui, A. Kolesnikov, S. Do, E.K. Bradley, J. Dotson, R. Goldsmith, T.P. Heffron, J. Lesnick, C. Lewis, J. Murray, J. Nonomiya, A.G. Olivero, J. Pang, L. Rouge, L. Salphati, B. Wei, C. Wiesmann, P. Wu, Cis-amide isosteric replacement in thienobenzoxepin inhibitors of PI3-kinase, Bioorg. Med. Chem. Lett. 23 (2013) 897-901.

[38] S. Pecchi, Z.-J. Ni, W. Han, A. Smith, J. Lan, M. Burger, H. Merritt, M. Wiesmann, J. Chan, S. Kaufman, M.S. Knapp, J. Janssen, K. Huh, C.F. Voliva, Structure guided optimization of a fragment hit to imidazopyridine inhibitors of PI3K, Bioorg. Med. Chem. Lett. 23 (2013) 4652-4656.

[39] P.N. Collier, G. Martinez-Botella, M. Cornebise, K.M. Cottrell, J.D. Doran, J.P. Griffith, S. Mahajan, F. Maltais, C.S. Moody, E.P. Huck, T. Wang, A.M. Aronov, Structural basis for isoform selectivity in a class of benzothiazole inhibitors of phosphoinositide 3-kinase gamma, J. Med. Chem. 58 (2015) 517-521.

[40] F. Giordanetto, B. Barlaam, S. Berglund, K. Edman, O. Karlsson, J. Lindberg, S. Nylander, T. Inghardt, Discovery of 9-(1-phenoxyethyl)-2-morpholino-4-oxo-pyrido[1,2-a]pyrimidine-7-carboxamides as oral PI3Kβ inhibitors, useful as antiplatelet agents, Bioorg. Med. Chem. Lett. 24 (2014) 3936-3943.

[41] T.D. Cushing, X. Hao, Y. Shin, K. Andrews, M. Brown, M. Cardozo, Y. Chen, J. Duquette, B. Fisher, F. Gonzalez-Lopez de Turiso, X. He, K.R. Henne, Y.-L. Hu, R. Hungate, M.G. Johnson, R.C. Kelly, B. Lucas, J.D. McCarter, L.R. McGee, J.C. Medina, T. San Miguel, D. Mohn, V. Pattaropong, L.H. Pettus, A. Reichelt, R.M. Rzasa, J. Seganish, A.S. Tasker, R.C. Wahl, S. Wannberg, D.A. Whittington, J. Whoriskey, G. Yu, L. Zalameda, D. Zhang, D.P. Metz, Discovery and in vivo evaluation of (S)-N-(1-(7-fluoro-2-(pyridin-2-yl)quinolin-3-yl)ethyl)-9H-purin-6-amine (AMG319) and related PI3Kδ inhibitors for inflammation and autoimmune disease, J. Med. Chem. 58 (2015) 480-511.

[42] Y. Shin, J. Suchomel, M. Cardozo, J. Duquette, X. He, K. Henne, Y.-L. Hu, R.C. Kelly, J. McCarter, L.R. McGee, J.C. Medina, D. Metz, T. San Miguel, D. Mohn, T. Tran, C. Vissinga, S. Wong, S. Wannberg, D.A. Whittington, J. Whoriskey, G. Yu, L. Zalameda, X. Zhang, T.D. Cushing, Discovery, Optimization, and in Vivo Evaluation of Benzimidazole Derivatives AM-8508 and AM-9635 as Potent and Selective PI3Kδ Inhibitors, J. Med. Chem. 59 (2016) 431-447.

[43] W.J. Scott, M.F. Hentemann, R.B. Rowley, C.O. Bull, S. Jenkins, A.M. Bullion, J. Johnson, A. Redman, A.H. Robbins, W. Esler, R.P. Fracasso, T. Garrison, M. Hamilton, M. Michels, J.E. Wood, D.P. Wilkie, H. Xiao, J. Levy, E. Stasik, N. Liu, M. Schaefer, M. Brands, J. Lefranc, Discovery and SAR of Novel 2,3-Dihydroimidazo[1,2-c]quinazoline PI3K Inhibitors: Identification of Copanlisib (BAY 80-6946), Chem. Med. Chem. 11 (2016) 1517-1530.

[44] S.L. Degorce, B. Barlaam, E. Cadogan, A. Dishington, R. Ducray, S.C. Glossop, L.A. Hassall, F. Lach, A. Lau, T.M. McGuire, T. Nowak, G. Ouvry, K.G. Pike, A.G. Thomason, Discovery of Novel 3-Quinoline Carboxamides as Potent, Selective, and Orally Bioavailable Inhibitors of Ataxia Telangiectasia Mutated (ATM) Kinase, J. Med. Chem. 59 (2016) 6281-6292.

[45] T. Bohnacker, A.E. Prota, F. Beaufils, J.E. Burke, A. Melone, A.J. Inglis, D. Rageot, A.M. Sele, V. Cmiljanovic, N. Cmiljanovic, K. Bargsten, A. Aher, A. Akhmanova, J.F. Díaz, D. Fabbro, M. Zvelebil, R.L. Williams, M.O. Steinmetz, M.P. Wymann, Deconvolution of Buparlisib's mechanism of action defines specific PI3K and tubulin inhibitors for therapeutic intervention, Nat. Commun. 8 (2017) 14683-14683.
